# Modeling CLN3 Batten disease in astrocytes reveals alterations in mitochondria homeostasis, fatty acid metabolism and oxidative stress response

**DOI:** 10.1186/s12929-026-01253-y

**Published:** 2026-05-13

**Authors:** Mingyi Yang, Wei Wang, María Cámara-Quílez, Borghild Hvesser Farsund, Niklas Nonboe Andersen, Karin Garten, Animesh Sharma, Xiaolin Lin, Ingrid Åmellem, Erlend Ravlo, Jing Ye, Magnar Bjørås, Mirta Mittelstedt Leal de Sousa

**Affiliations:** 1 Department of Microbiology, Oslo University Hospital, Oslo, Norway; 2 Department of Clinical and Molecular Medicine, Norwegian University of Science and Technology, NTNU, Trondheim, Norway; 3 Proteomics and Modomics Experimental Core Facility (PROMEC) at NTNU, and the Central Norway Regional Health Authority, Trondheim, Norway; 4 Centre for Embryology and Healthy Development, University of Oslo, 0373 Oslo, Norway; 5 Department of Medical Biochemistry, Oslo University Hospital, Oslo, Norway

**Keywords:** CLN3 Batten disease, CLN3 patient-derived astrocytes, Mitochondrial function, Lipid metabolism, Oxidative stress response

## Abstract

**Background:**

CLN3 Batten disease is a severe pediatric neurodegenerative disorder caused by mutations in the *CLN3* gene, most commonly a 1 kb deletion encompassing exons 7 and 8. CLN3 deficiency is associated with lysosomal dysfunction, impaired cellular clearance and disrupted metabolism. While neurons are particularly vulnerable in CLN3 Batten disease and have been the primary focus of research, glial cells are increasingly recognized as active contributors to disease pathology. Among them, astrocytes—the most abundant glial cell type in the brain—play critical roles in maintaining neuronal health and homeostasis. However, astrocytes remain understudied in CLN3 patient-derived models.

**Methods:**

We present the first iPSC-derived astrocyte model from a skin biopsy of a CLN3 patient carrying the common 1 kb deletion. Cellular and molecular features of iPSC and astrocytes derived from both healthy controls and the CLN3 patient were characterized via qPCR, immunocytochemistry and targeted mass spectrometry. In addition, comprehensive omics-based profiling, through transcriptomic and label-free quantitative proteomics, was performed to uncover novel molecular mechanisms and generate hypotheses that can guide future mechanistic and functional studies.

**Results:**

Transcriptomic and proteomic analyses during astrocyte differentiation revealed an upregulation of mitochondrial respiratory chain complexes I and IV—contrasting with the downregulation typically observed in CLN3-deficient neurons. We also identified a metabolic shift favoring the elongation of very-long-chain saturated fatty acids, accompanied by reduced lipid synthesis and enhanced fatty acid oxidation. These metabolic alterations were paralleled by an upregulation of proteins involved in oxidative stress responses, likely reflecting a compensatory adaptation to mitochondrial and lipid metabolic dysregulation. Furthermore, we observed significant changes in chromatin organization during astrocyte differentiation in CLN3 cells, suggesting epigenetic remodeling as a contributing factor to disease pathology.

**Conclusion:**

Our findings prompt the hypothesis that mitochondrial dysfunction may precede lysosomal defects in CLN3-deficient astrocytes. Restoring mitochondrial health could improve brain metabolism, inflammation control, neurotransmitter regulation, and neuronal survival, highlighting mitochondria as promising therapeutic targets in CLN3 Batten disease.

**Supplementary Information:**

The online version contains supplementary material available at 10.1186/s12929-026-01253-y.

## Introduction

Batten Disease, or Neuronal Ceroid Lipofuscinosis (NCL), constitutes a family of devastating lysosomal storage disorders (LSDs), caused by mutations in at least one of 13 genes, and collectively represent the most common inherited pediatric neurodegenerative disorder worldwide [1, 2]. Globally, the prevalence of Batten disease is 1 in 100,000 live births [3], and the most common form of NCL is CLN3 Batten disease, also known as juvenile neuronal ceroid lipofuscinosis (JNCL). CLN3 Batten disease is caused by mutations in the *CLN3* gene, and the most common abnormality observed in CLN3 patients is the loss of exons 7 and 8, also called the 1 kb deletion. The neurological symptoms of the disease usually manifest between 4 and 12 years of age with initial signs of vision loss that progress to blindness, cognitive impairment, motor decline and onset of seizures, as well as brain atrophy culminating in premature death between 15 to 30 years of age [4]. At the cellular level, predominant features of CLN3 Batten disease include retinal degeneration, neuronal loss, glial reactivity, and accumulation of intracellular autofluorescent storage material (ASM), whose major protein component is the subunit C of mitochondrial ATP synthase (SCMAS) [5–7]. Animal models also showed impaired neurodevelopment [8].

The *CLN3* gene encodes a transmembrane protein primarily expressed in endosomes and lysosomes. The CLN3 protein has been associated with cellular homeostasis and neuronal survival, but its precise functions remain unknown. In mice, CLN3 was shown to be highly abundant in neuronal cells, localizing to axonal extensions and synaptosomes, suggesting a role in synapses regulation [9]. Recently, disrupted glycerophospholipid catabolism was reported in lysosomes of brain cells in *Cln3*^*−/−*^ mice [10]. As *CLN3* mutation severely affects neurons, molecular mechanisms underlying neuronal cell death due to CLN3 loss and its role in neuronal cell differentiation have been extensively studied [9, 11–15]. Over the last decades, however, the traditional “neuron-centric” conception of NCL has been reassessed, as an increasing body of evidence pinpoints that glial cell dysfunction contributes to NCL pathology. For instance, early glial activation was shown to precede neuron loss in *Cln3*^−/−^ mice [16, 17]. Accurate prediction of where the subsequent selective neuron loss will occur based on glial activation has also been reported in mouse models of other NCL subtypes [18]. Moreover, in vitro co-culture experiments revealed that *Cln3*^−/−^ glia are detrimental to the survival and affect the morphology of both wild-type and mutant neurons [19]. Importantly, the co-culture of mutant neurons with healthy glial cells largely rescued these effects, suggesting that glial cells may be functionally compromised and possibly actively contribute to neurodegeneration in CLN3 Batten disease.

Glial cells comprise astrocytes, microglia, and oligodendrocytes, with astrocytes being the most abundant glial cell type in the brain. These cells play critical roles in neuronal homeostasis and neurodegeneration [20]. Upon infection, brain injury, and neurodegenerative diseases, astrocytes and microglia become activated and mediate neuroimmune responses by altering their protein expression and secretion profile [21, 22]. Astrocytes also exert non-inflammatory functions, including nutrient supply to neurons, extracellular ion balance maintenance, antioxidative support, growth factors release, and synchronizing neurotransmitter metabolism and release. Notably, it was recently shown that astrocytes play a key role in refining synaptic connectivity via phagocytosis [23–25] and that impairment of astrocytic engulfment of synapses affects the visual system [25]. Furthermore, astrocytes are key cell types promoting mitochondrial shift to provide neuroprotection to the central nervous system. In this context, healthy mitochondria from astrocytes have been shown to be transferred to injured neurons for detoxification [26]. Conversely, damaged mitochondria from dopaminergic neurons can also be transferred to astrocytes for clearance via transneuronal mitophagy, a mechanism implicated in Parkinson’s disease [27]. Thus, due to the prominent role of astrocytes in human disease, establishing appropriate disease models to investigate cell-type-specific contributions to pathogenesis and to explore potential treatment modalities is of utmost importance.

Recent advances in stem cell technologies provide a remarkable new tool to study the unique and dynamic features of human brain development and neurological disorders. The human induced pluripotent stem cells (iPSCs) derived from patient skin cells such as fibroblasts, can be differentiated into brain cells in vitro, enabling a detailed cell-type-specific study of the pathogenesis of inherited brain diseases. To date, several patient-specific iPSC-derived models have been established for CLN3, including neural progenitor cells [28], blood–brain barrier [29], retinal pigmented epithelium [30], cerebral organoids [31] and cortical neurons [15]. However, no iPSC-derived glial models have so far been generated to study this disease. Here, we generated a CLN3 patient-derived astrocyte model via reprogramming of fibroblasts from skin biopsies of a patient carrying the 1 kb deletion in the *CLN3* gene. To elucidate biological networks affected by loss of CLN3 during glial differentiation, we conducted global transcriptome and proteome analyses of iPSCs and astrocytes derived from healthy controls and the CLN3 patient. We found marked dysregulation of proteins involved in mitochondria-related processes, particularly proteins belonging to the mitochondrial respiratory complexes I and IV. Moreover, we found a shift in fatty acid metabolism in the CLN3 deficient astrocytes, potentially leading to the accumulation of long chain lipids and upregulation of fatty acid oxidation, as well as an upregulation of proteins involved in the response against oxidative stress. This study provides a comprehensive atlas of genes, proteins, biological processes, and pathways altered by CLN3 deficiency during astrocytogenesis. Serving as a foundational resource for the field, it offers a rich dataset to inform and prioritize future mechanistic and functional studies, with the potential to guide the development of novel therapeutic strategies for CLN3 Batten disease.

## Methods

### Patient samples and iPSC reprogramming

A skin biopsy from male CLN3 patient homozygous for the canonical 1 kb deletion in the CLN3 gene was obtained in Trondheim, Norway 2019. Informed consent for participation in the study was provided by the parents of the CLN3 child. Healthy control fibroblast lines (two cell lines) were obtained from ATCC (cat. number PCS-201—012) and the Coriell Institute (cat. number AG05836). Fibroblasts from the skin biopsy and healthy controls were cultured at 37 °C with 5% CO2 in growth medium consisting of DMEM medium, supplemented with 10% fetal bovine serum, 100 IU/mL penicillin and 100 μg/mL streptomycin. Fibroblasts were then reprogrammed into iPSC using CytoTune®-iPS 2.0 Sendai Reprogramming Kit (Invitrogen) using a multiplicity of infection of 5:5:3 (KOS:c-Myc:Klf4) according to the manufacturer’s instructions. The iPSCs were maintained and expanded on Geltrex coated 6-well plates with essential 8 medium.

### Generation of astrocytes

**Fig. 1:**
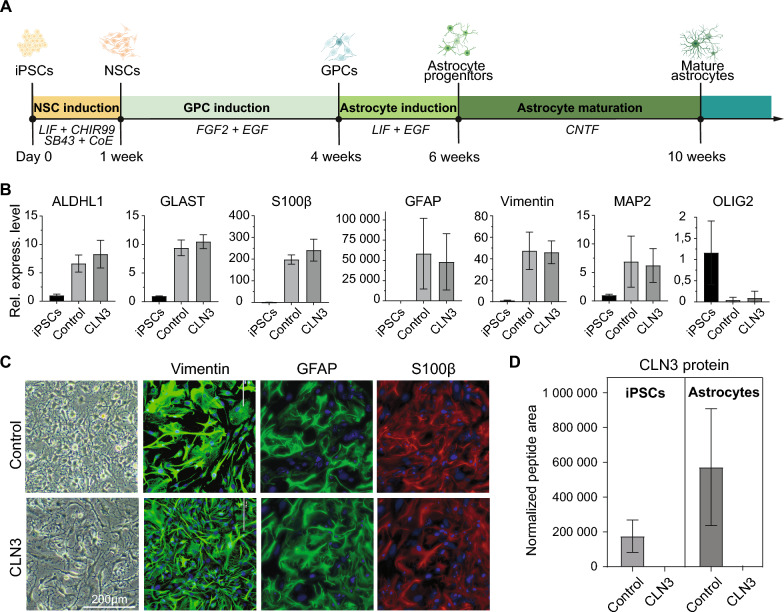
Generation and characterization of iPSC-derived astrocytes. **A** Schematic overview of astrocyte differentiation from patient-derived iPSCs. Key compounds used to drive differentiation toward a mature astrocyte phenotype are indicated: LIF (leukemia inhibitory factor), CHIR99021, SB431542, CoE, FGF2 (fibroblast growth factor 2), EGF (epidermal growth factor), and CNTF (ciliary neurotrophic factor). **B** qPCR analysis of astrocyte-specific markers (ALDHL1, GLAST, S100b, GFAP, Vimentin) in iPSC-derived cells. Expression of MAP2 (neuron marker) and OLIG2 (oligodendrocyte marker) was assessed to evaluate cell population purity. **C** Phase-contrast images and immunocytochemical validation of astrocyte marker expression. Vimentin and GFAP (green), S100β (red), and DAPI-stained nuclei (blue) are shown. **D** Quantification of CLN3 protein levels in control and CLN3 patient-derived iPSC and astrocytes via targeted mass spectrometry

The iPSCs were first differentiated into neural stem cells (NSCs) and further into glial progenitor cells (GPCs) following the protocols by Li et al. [32]. Subsequent differentiation of GPC into astrocytes was performed based on protocols from Perriot et al. [33], with minor modifications (Fig. 1A). Briefly, iPSCs at ~ 70—80% confluency were lifted with 0.5 mM EDTA and plated onto Geltrex-coated 6-well plates in Essential 8 medium with 10 μM Y-27632. On the second day, the medium was switched to neural induction medium (50% of each DMEM/F12/GlutaMax and Neurobasal A, 1 × GlutaMax, 50 ug/ml BSA, 1 × N2 supplement, 1 × B27 supplement without Vitamin A, 10 ng/ml human LIF, 4 μM CHIR99021, 3 μM SB431542, 0.1 μM Compound E and 10 μM Y-27632). The medium was changed every day for 7 days. The induced neural progenitor cells were dissociated with accutase, passaged at 1:3 ratio and cultured for another 7 days with the neural induction medium without compound E. Afterwards, the cells were passaged and cultured for 6—8 passages in glial progenitor medium consisting of DMEM/F-12 + Glutamax, 1 × N2 supplement, 1 × B27 without vitamin A, 1% fetal bovine serum, 10 ng/ml FGF-2 and 10 ng/ml EGF. At the passage 7 and 8, cultured glial progenitor cells were plated onto Geltrex-coated plates at 80,000 cells/cm2 in astrocyte induction medium (DMEM/F-12/GlutaMax, 1 × N2 supplement, 1 × B27 without vitamin A, 10 ng/ml EGF, and 10 ng/ml LIF) for 14 days. The maturation of astrocytes was induced with astrocyte maturation medium (DMEM/F-12/Glutamax, 1 × B27 without vitamin A, 20 ng/ml CNTF) for 4 weeks.

### Cell characterization via quantitative real-time polymerase chain reaction (qPCR)

Total RNA was isolated from patient and control samples using the RNeasy Kit (Qiagen) following the manufacture’s protocol. For RT-qPCR, Complementary DNA (cDNA) was generated from total RNA samples using the High-capacity cDNA reverse transcription kit (Applied Biosystems, 4,368,814) according to the manufacturer’s instructions. Real-time PCR reactions were performed using Power SYBR Green Master mix in the StepOnePlusTM Real-Time PCR System (Applied Biosystem) with the standard cycle conditions. Samples were measured in triplicates. ß-actin and GAPDH were used as internal references. The ΔΔCT method was used to quantify the relative gene expression. Information on primer sequences is provided in Table 1.

**Table 1:** Nucleic acid sequence (5’ à 3’) of primers used during qPCR analysis

Gene	Forward primer	Reverse primer	Cell type marker
*NANOG*	AATACCTCAGCCTCCAGCAGATG	TGCGTCACACCATTGCTATTCTTC	iPSC
*OCT4*	GTACTCCTCGGTCCCTTTCC	CAAAAACCCTGGCACAAACT	iPSC
*SOX2*	GAGCTTTGCAGGAAGTTTGC	GCAAGAAGCCTCTCCTTGAA	iPSC
*MAP2*	CAGGGACACCAACCTTGACT	CTCCTAACACTCCGGCTCTG	Neuron/active astrocytes
*OLIG2*	GCTGCGTCTCAAGATCAACA	CACCAGTCGCTTCATCTCCT	Oligodendrocyte
*GFAP*	ACCTCCTCCTCGTGGATCTT	GAAGCTCCAGGATGAAACCA	Astrocytes
*GLAST*	AGCAGGGAGTCCGTAAACG	AGCATTCCGAAACAGGTAACTTT	Astrocytes
*S100β*	CCAGCCGTGTTGTAGCTAAT	CAGCTTACACACAGGCCTAATA	Astrocytes
*ALDHL1*	CGCTGTACAACCGCTTCCTC	CCTGCACCATCCCTTTGATG	Astrocytes
*VIMENTIN*	GAGAACTTTGCCGTTGAAGC	TCCAGCAGCTTCCTGTAGGT	Astrocytes/fibroblasts
*GAPDH*	GAAGCTCCAGGATGAAACCA	ACCTCCTCCTCGTGGATCTT	(Housekeeping gene)

### Cell characterization via immunocytochemistry

iPSC and astrocytes were fixed with 4% paraformaldehyde for 15 min, permeabilized with 0.1% Triton-X100 and blocked with 5% bovine serum albumin, 5% normal goat serum, and 0.1% Triton X-100 at room temperature for 30 min. For immunocytochemistry, the blocked cells were incubated with primary antibodies Nanog (Cell Signaling 4903S, 1:500), OCT-4A (Cell Signaling 2840S, 1:500), TRA-1—60 (Cell Signaling 4764S, 1:200), CXCR4 (Stemcell Technologies 60,089, 1.500), SOX17 (R&D Systems AF2085, 1:200, Brachyury (R&D Systems AF2085, 1:500), GFAP (Dako Z0334, 1:1000), S100β (Sigma HPA015768, 1:500) and vimentin (Thermo Fisher, MA5—1188, 1:500) overnight at 4⁰C. After washing with 0.1% Tween-20/PBS, the samples were incubated with Alexa Fluor 594 and Alexa Fluor 488 conjugated secondary antibodies (ThermoFisher, 1:500 dilution) for 1 h and counterstained with DAPI nuclear staining. Images were acquired using fluorescence microscope EVOS FL Auto (Thermo Fisher Scientific). For confocal microscopy analysis, the cells were incubated with rabbit anti-ATP synthase subunit C (ATPsynC) (Abcam, ab181243, 1:100) overnight at 4 °C followed by incubation with fluorescent-conjugated secondary antibody Alexa Fluor 555 (Thermo Fisher Scientific, A27039, 1:1000) for 1 h. ProLong™ Gold Antifade Mountant with DAPI (#P3693, Thermo Fisher Scientific, Inc., Waltham, MA, USA) was added to the samples and analyzed using Zeiss LSM 880 Confocal Microscope.

### Western-blot analysis

**Fig. 2:**
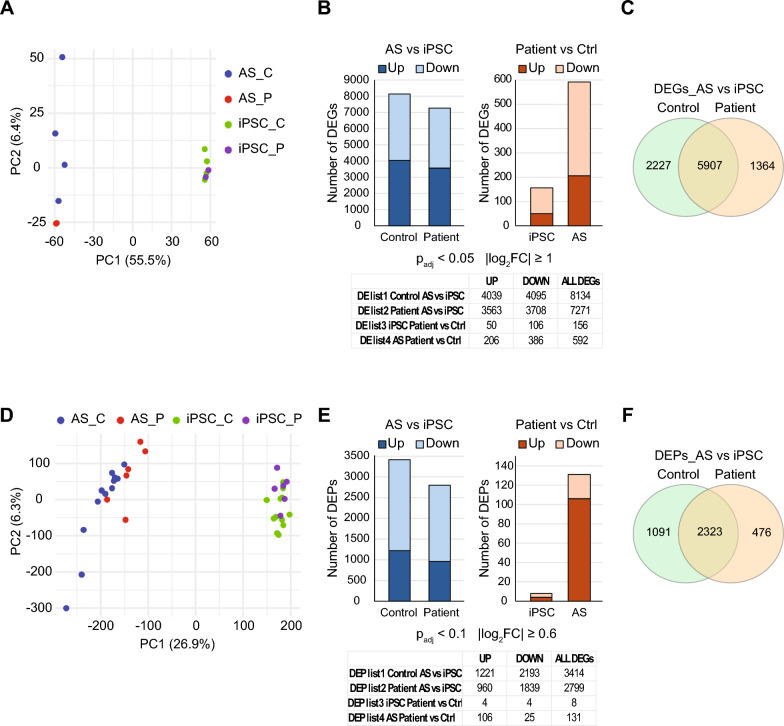
Comparison between overall changes in RNA transcripts and proteins throughout astrocyte differentiation. **A** Principal component analysis (PCA) of RNA sequencing (RNAseq) data reveals distinct clustering of cell types. Each point represents one sample consisting of a pool of three replicates, color-coded by cell type and genotype. The first two principal components (PC1 and PC2) are shown, accounting for the largest variance in the dataset. Distinct clustering of samples indicates transcriptional differences between cell types, supporting the identity and separation of the populations analyzed. **B** Bar plots showing the total number of differentially expressed genes (DEGs) during astrocyte differentiation and at specific developmental stages (iPSC and astrocytes) using a threshold of Log2Fold change ≥ 1 and p-adjusted values < 0.05. The proportions of upregulated and downregulated genes are represented in dark and light colors, respectively. **C** Venn diagrams illustrate the number of unique and overlapping DEGs in control and CLN3 patient groups during differentiation. **D** PCA of proteome data. Each dot represents an individual sample, color-coded by cell type and genotype. **E** Bar plot representation of global proteome alterations during astrocyte differentiation and at stage specific comparisons using a threshold of Log2Fold change ≥ 0.6 and p-adjusted values < 0.1. The proportion of differentially expressed proteins (DEPs) follow the same color code adopted for DEGs. **F** Venn diagrams illustrate the number of unique and overlapping DEPs in control and CLN3 patient groups during differentiation

Protein lysates (50 μg) were heated in 1 × LDS loading buffer (Invitrogen) containing 1 mM DTT for 10 min at 70 °C prior to separation on pre-cast 4–12% NuPAGE gel using MES running buffer (50 mM MES (2-[N-morpholino]ethanesulfonic acid), 50 mM Tris base, 1 mM EDTA, 0.1% (w/v) SDS) at 200 V for 1 h. Proteins were then transferred to mini 0.2 µm PVDF membranes (BIO-RAD) using a semi-dry trans-blot Turbo system (BIO-RAD) following the manufacturer’s instructions. In Fig. 2C, Western blotting analysis were performed using rabbit anti-ATP synthase subunit C (ATPsynC) (Abcam, ab181243, 1:1000) and mouse monoclonal actin antibody for data normalization (Sigma, A5441, 1:1000) as primary antibodies. After incubation with IRDye® 800LT goat anti-mouse and IRDye® 680LT goat anti-rabbit IgG Secondary Antibodies (Li-COR), bands were detected on an Odyssey infrared imaging system (Li-COR) and quantitative analysis performed by the Image studio software version 2.1.15. The average SCMAS intensity levels were quantified in three patients and two healthy controls at the iPSC and astrocyte stage. The difference in intensity level between the patient and control group was tested using the two-tailed two-sample t-test.

### Determination of mitochondrial copy number

The qPCR was performed with StepOnePlus real time PCR system to determine the relative mtDNA copy number by comparing ratio of mtDNA to nuclear DNA. Oligonucleotide primers used were: mitochondrial gene mt-Rnr1 forward, 5’-AAACTGCTCGCCAGAACACT; mt-Rnr1 reverse, 5’-CATGGGCTACACCTTGACCT; nuclear gene human β-globin (HBB) forward, 5’-GAAGAGCCAAGGACAGGTAC; HBB reverse, 5’-CAACTTCATCCACGTTCACC. Relative standard curves were established by plotting the threshold value versus the log of serial dilutions of total DNA added to the reaction according to the protocol described in Bulletin #2 (Applied Biosystems, CA). Relative mtDNA copy numbers were calculated based on the standard curve and the ratio of the amount of mtDNA versus nuclear gene for each sample. The values were normalized to the amount of mtDNA and HBB of undifferentiated iPSC and converted into fold changes.

### Transcriptome analysis

#### Sample preparation

iPSC and astrocytes were collected from two clones derived from two healthy control individual (total number of clones = 4) and two clones derived from a CLN3 child (total number of clones = 2). For each clone, the differentiation procedure was carried out three times. For transcriptome analysis, each sample consists of a pool of three replicates collected from independent differentiation experiments, both for iPSC and astrocyte cell types. RNA was extracted using the RNeasy Mini Kit from Qiagen according to the manufacturer's instructions. RNA samples typically yielded > 100 ng of RNA with a RIN value of > 7 as determined by Bioanalyzer (Agilent Technologies). Whole-transcriptome sequencing was performed by BGI Genomics Co., Ltd., Hong Kong, China using the DNBSEQ Eukaryotic Strand-specific Transcriptome Resequencing platform with PE100, with at least 30 M clean reads per sample (circa 6Gb clean data per sample). The clean data was provided by BGI and the bioinformatic analysis was performed in our lab.

#### Data processing and analysis

The sequencing raw data (fastq file) contained 40 million paired reads per sample with 100 bp per read in length. The raw data was cleaned, and the quality control was performed by FastQC and MutiQC. The clean paired-reads were aligned to human reference genome hg38 and transcriptome gtf files (download from https://genome-idx.s3.amazonaws.com/hisat/hg38_tran.tar.gz). The sequence alignment was performed using HISAT2 package in a clustering computer of Saga provided by UNINETT Sigma2 (Trondheim, Norway). The aligned data of sam files were sorted and transferred into bam files by SAMtools. The transcripts from all samples were assembled using stingTie (v1.3.5) and merged by stringTie –merge function. The read coverage tables including normalized counts in FPKM unit were generated based on the merged transcripts using stringTie –eB function. The DESeq2 tool (R package, V1.30.1) was used to compare all transcripts of the read coverage tables, normalize counts between conditions and identify the differentially regulated transcriptions in gene level (Differentially Expressed Genes, DEGs). In DeSeq2, the raw count was normalized by the size factor, which is the median of ratios. The ratio for each gene was calculated by raw_count/geometric_mean, while the geometric mean is defined as the geometric mean value in observed raw_counts cross all samples in a gene. A modified t-test (Wald test) was used in statistical p value analysis. The DEGs were defined as absolute log2 fold change >  = 1 and adjusted p-value < 0.05. The DEGs were further processed and visualized using R packages, including ggplot2, tidyverse, PCA, ggvenn (v.0.1.9, for venn diagram) and pheatmap (V.1.0.12). Gene Ontology (GO) analysis was performed as described below.

### Mass spectrometry analysis

#### Sample preparation

Two clones of each healthy control individual (n = 2) and CLN3 child (n = 1) were reprogrammed into astrocytes three times, resulting in a total of 12 healthy control samples and 6 CLN3 patient samples per stage of differentiation (iPSC, astrocytes). Cell pellets were reconstituted in RIPA buffer (150 mM NaCl, 50 mM Tris–HCl pH 7.4, 1% NP40, 0.5% sodium deoxycholate, 0.1% SDS, 10 mM EGTA, 10 mM MgCl_2_,) containing 1% phosphatase inhibitor cocktails 1 and 3 (Sigma), 2% Complete EDTA-free (Roche) and a nuclease cocktail composed by Benzonase (≥ 250 units/µL, Millipore), Micrococcal nuclease (229 units/µL Thermo Scientific) and RNase (10 mg/ml, Sigma-Aldrich) added at 1 µL per 10 mL RIPA buffer. Protein concentration of supernatants was determined by using the BioRad protein assay (BioRad laboratories). Automated protein purification and trypsin digestion were performed using a KingFisher Flex system (Thermo Scientifics) using 96 deep-well plates. For each sample, 25 μg protein were mixed with SDC buffer (1% Sodium deoxycholate, 10 mM TCEP (tris(2-carboxyethyl)phosphine)), 40 mM Chloroacetamide, 100 mM Tris–HCl pH8.5), for simultaneous protein reduction and alkylation for 30 min. Protein binding to MagReSyn HILIC microspheres (20 μg, resyn biosciences), which were pre-equilibrated in 15% acetonitrile (ACN) 100 mM ammonium acetate (NH_4_Ac) pH 4.5 buffer, was performed in binding buffer (0.2% SDS, 30% ACN, 200 mM NH_4_Ac). After washes in 95% ACN, on bead trypsin digestion was performed in 15 mM NH_4_HCO_2_ pH 8.2 containing 5 ng/μL trypsin protease MS-grade (Thermo Scientific) for 6 h at 37 °C. Samples were dried out in a speed vac, reconstituted in 45 μL of 0.1% FA, centrifuged for 30 min at 16,000 g and 4 °C, and supernatants were transferred to HPLC vials.

#### Targeted mass spectrometry

All parallel reaction monitoring (PRM)-based targeted mass spectrometry methods were designed, analyzed, and processed using Skyline software version 23.1.0.455 [34]. In silico selection of proteotypic peptides was performed via Skyline using the Homo sapiens reference proteome available at www.uniprot.org to exclude non-unique peptides. Heavy labelled peptide standards were first analyzed on a Thermo Scientific Q Exactive HF mass spectrometer operating in PRM mode. This data was imported into skyline and used for the selection of the top ionizing peptides (2 + and 3 + charge states) and to build a scheduled method with retention time windows of 10 min. The method was then employed for detection and quantification of corresponding peptides in the samples. Here, information on retention time and fragmentation pattern of the heavy labelled peptide standards was used for peptide identification and chromatographic quality control. The same instrument parameters described below for sample analysis were adopted for establishment of the PRM method with standard peptides.

Tryptic digests (2 µg) together with synthetic peptide standards containing heavy labeled Lysine (+ 8) or Arginine (+ 10) (PEPotec SRM Grade 2 Peptides, Thermo scientific) (20 fmol) were analyzed on a Q Exactive HF mass spectrometer operating in PRM mode coupled to an EASY-nLC 1200 UHPLC system (Thermo Scientific). Peptides were injected onto an Acclaim PepMap C18 column (75 µm i.d. × 2 cm nanoviper, 3 µm particle size, 100 Å pore size) (Thermo Scientific) and further separated on an EASY Spray™ LC column (75 µm i.d. × 50 cm nanoviper, 2 µm particle size, 100 Å pore size) (Thermo Scientific) at 40 °C. The following 100 min method was used at 300 nl/min flow rate: starting with 6% solvent B (80% Acetonitrile, 0.1% Formic acid) with an increase to 35% solvent B in 81 min, followed by an increase to 100% solvent B over 9 min, where it was subsequently held for 10 min. Solvent A consisted of 0.1% Formic acid. The peptides eluting from the column were ionized by an Easy Spray™ Source (Thermo Scientific) and analysed on positive-ion mode using electrospray voltage 1.75 kV and HCD fragmentation. Each MS/MS scan was acquired at a resolution of 60 000 FWHM, normalized collision energy (NCE) 28, automatic gain control (AGC) target value of 2 × 10^5^, maximum injection time (mIT) of 110 ms and isolation window 1.4 m/z.

Peptide quantification was achieved by summing the integrated peak areas of the most intense fragments. Peptide areas for multiple peptides of the same protein were summed to assign relative abundance to that protein. Endogenous β-actin (uniport code: G8FSY7) levels were used for data normalization. To evaluate the levels of each protein in distinct groups, values of relative abundance of each protein in samples belonging to the same group (Control or CLN3) were averaged. The error bars represent standard deviation of biological replicates. For CLN3, peptides exclusively belonging to WT or mutant CLN3 sequences and peptides common to both WT and mutant CLN3 sequences, were included in the analysis. However, only one peptide, common to WT and mutant sequences, was detected in iPSCs and astrocytes samples derived from healthy controls. No signal was detected in CLN3 patient-derived cells.

#### Global proteome analysis

LC–MS/MS analysis was performed on a timsTOF Pro 2 (Bruker Daltonics) connected to a nanoElute (Bruker Daltonics) HPLC. Peptide separation was performed using a Bruker25 (150um*25 cm) column with running mobile phases A (0.1% formic acid) and B (0.1% formic acid in acetonitrile). The nano-flow LC gradient was delivered at 250 nL/min and consisted of a 120 min gradient starting with mobile phase B increasing from 2 to 17% B in 60 min, followed by increases to 25% B in 30 min, 37% in 10 min, and to 95% B in 10 min, where it was hold for 10 min. HPLC was coupled to MS by a Captiva Ion Source with a ZDV Sprayer with ID 20 um (Bruker Daltonics). The timsTOF instrument was operated in the DDA PASEF mode with 10 PASEF scans per acquisition cycle and accumulation and ramp times of 100 ms each. MS and MS/MS data were acquired over an m/z range of 100–1,700. The ‘target value’ was set to 20,000 and dynamic exclusion was activated and set to 0.4 min. The quadrupole isolation width was set to 2 Th for m/z < 700 and 3 Th for m/z > 800 [35, 36]. Database search was performed with MaxQuant.

#### Data processing and analysis

Open workflow [37] was used to inspect the raw data to determine optimal search parameters for MaxQuant v 2.0.3.0 [38]. Along with defaults, deamidation of asparagine/glutamine as dynamic post-translational modification was appended. Search performed against the Human proteome including isoforms downloaded from Uniprot (https://www.uniprot.org/proteomes/UP000005640) in June 2022 and MaxQuant’s internal contaminants database using Andromeda built into MaxQuant. Match-between-runs (MBR) functionality is utilized to reduce missing values in raw data. MBR verifies the presence of a peptide signal in one sample that wasn’t identified in another. This is achieved by utilizing high mass precision and a restricted retention time period of 1-min with 20-min overall sliding window. If this particular signal is detected (MS1 intensity threshold 30), it is labeled as ‘By matching’ instead of ‘By MS/MS’ in the output. Peptide group identifications false discovery rate was kept at 1% and only unique peptides with high confidence were used for downstream protein group identification.

The peptide intensity data was cleaned by removing samples with low peptide coverage (< 1000 peptides) and removing peptides with missing value in 90% of samples in at least one group. The data was further log-transformed, normalized by centralizing the median value of peptides among samples. The normalized peptide data was aggregated into protein level (by “robustSummary” method in the function of “aggregateFeatures”) using a R package Qfeatures (v 1.12.0), according to Sticker, A. et al. [39]. The differentially expressed proteins (DEPs) were identified by a linear regression model in limma package (v 3.58.1). The default setting for DEPs was by log2FoldChange > 1 and adjusted p_value < 0.05. Because low number of DEPs were identified by the default setting (only 32 DEPs in the comparison of patient vs control in astrocyte), we optimized the DEPs setting by absolute log2FoldChange > 0.6 and adjusted p_value < 0.1.

### Gene ontology and pathway analysis

In both RNA-seq and proteomics data, the gene ontology over-representation (GO over-representation) analysis, gene set enrichment analysis (GSEA) for GO (GSEA-GO) and for Kyoto Encyclopedia of Genes and Genomes (GSEA-KEGG) were performed by a R package of clusterProfiler (v 4.10.1) in default setting. The KEGG pathway was viewed by R package of pathview (v 1.42.0).

### Statistical analysis

The linear regression module of limma was used to identified differentially expressed proteins between paired groups. The adjusted p-value was calculated by Benjamini-Hochberg (BH) approach in enrichment analysis of GO, KEGG pathway and GSEA.

## Results

### Generation and characterization of CLN3 patient-derived iPSC and astrocytes

Astrocyte generation begins with the reprogramming of fibroblasts, obtained from skin biopsies of a CLN3 patient and healthy control individuals, into induced pluripotent stem cells (iPSCs). To evaluate the quality of the generated iPSC, pluripotency features were assessed via cell morphology, expression of pluripotency markers and trilineage differentiation capacity (Supplementary Fig. 1). The iPSCs exhibited the expected characteristic morphological features, forming colonies with distinct edges and displaying large nuclei centrally located in cells of sparse cytoplasm (Supplementary Fig.  1 A). A marked increase in expression of the pluripotency markers was observed in the iPSC compared to fibroblasts at the mRNA and protein levels (Supplementary Figs. 1B and 1 C). No significant differences in morphology and expression patterns of the pluripotency markers were observed between control and CLN3-patient derived iPSC. Pluripotency capacity was evaluated via iPSC differentiation into mesoderm and endoderm germ layers (Supplementary Fig. 1D). Capacity to ectoderm differentiation was proved upon successful differentiation into astrocytes.

The iPSCs were differentiated into neural progenitor cells (NPC), based on protocols described by Li et al. [32], and the NPC were further differentiated into glial progenitor cells (GPC) and astrocytes following the method described by Perriot et al. [33] (Fig. 1A). The identity of the iPSC-derived mature astrocytes was confirmed at the molecular level via qPCR and ICC (Fig. 1B and C). High expression of GFAP and a > 100-fold increase in S100B, both markers of mature astrocytes, were observed. Elevated levels of ALDH1 and EAAT1/GLAST further supported astrocyte maturation, while reduced vimentin expression indicated a low proportion of immature cells. To assess population purity, MAP2 and OLIG2 expression were evaluated. OLIG2 expression level was higher in iPSCs than in the differentiated cells, suggesting minimal oligodendrocyte contribution in the latter. Low MAP2 expression may reflect either minor neuronal presence or astrocytic activation. Together, these data confirm successful differentiation of control and CLN3 iPSCs into astrocytes.

About 85% of all CLN3 patients, including the patient participating in this study, are homozygous for the 1 kb deletion [40]. To evaluate whether the cells preserved their phenotype throughout reprogramming and differentiation, *CLN3* mRNA and protein levels were measured by qPCR and parallel reaction monitoring (PRM), a targeted mass spectrometry approach, respectively. qPCR confirmed the expression of two mutated *CLN3* alleles in the iPSC patients, and two normal *CLN3* alleles in the iPSC from healthy controls (Supplementary Fig. 1E). This data confirms that the iPSCs had preserved their genotype throughout reprogramming. Additionally, PRM confirmed the expression of CLN3 in the healthy control-derived iPSC and astrocytes. Conversely, the truncated CLN3 protein was not detected in CLN3-derived cells (Fig. 1D). This data indicates that the deletion of exons 7 and 8 in the CLN3 gene abrogates the expression of the mutant CLN3 protein.

### Integrated transcriptomic and proteomic profiling reveals CLN3-dependent alterations during astrocyte differentiation

To dissect the impact of CLN3 mutation on astrocyte differentiation, we combined developmental trajectory and stage-specific analyses of transcriptomic and proteomic data. Developmental comparisons across the iPSC-to-astrocyte transition reveal how the mutation disrupts dynamic regulatory programs essential for lineage progression and maturation. In contrast, stage-specific analyses enable the identification of mutation-specific signatures that may not be evident in developmental comparisons, such as baseline dysregulation in pluripotent cells or astrocyte-specific functional impairments. Together, these approaches provide a comprehensive view of both temporal and cell-state-specific effects of CLN3 dysfunction during astrocyte development.

In the first approach, the developmental trajectory of control and CLN3 patient-derived cells from the initial (iPSC) to final (astrocyte) differentiation stages were analyzed via RNA sequencing (RNA-seq) and label-free quantitative shotgun mass spectrometry. Principal components analysis (PCA) revealed distinct clusters for iPSCs and astrocytes, independent of the genotype, suggesting a well-defined, stage-specific glial differentiation process both at mRNA and protein levels (Fig. 2A and D). A total of 8134 differentially expressed genes (DEGs) were identified in the control group throughout differentiation from iPSC to astrocytes, while 7271 DEGs were identified in the CLN3 patient group, with similar number of up- and down-regulated genes in both genotypes (Fig. 2B, left panel). Of these, 5907 DEGs are common between control and patient groups, while 2227 and 1364 DEGs are unique to the control and CLN3 patient groups, respectively (Fig. 2C).

For the proteome analysis, 3414 and 2799 differentially expressed proteins (DEPs) were identified in the control and patient groups, respectively, throughout differentiation. A higher number of downregulated proteins were detected in both control and CLN3 patient groups (Fig. 2E, left panel). 2323 common DEPs were identified between the two genotypes, while 1091 and 476 DEPs were uniquely identified in the control and patient groups, respectively (Fig. 2F). Volcano plots are provided in Supplementary Fig. 2A. Lists of DEGs and DEPs identified throughout differentiation are provided in Supplementary Tables 1A-B and 2A-B, respectively.

For developmental stage-specific comparisons between control and CLN3 patient-derived cells, we identified 156 DEGs at the iPSC stage and 592 DEGs at the astrocyte stage (Fig. 2B, right panel). Lists of DEGs and DEPs identified in stage-specific comparisons are provided in Supplementary Tables 1C-D and 2C-D, respectively. A higher number of down-regulated DEGs were observed at both stages. At protein level, 8 DEPs were identified at iPSC stage (Fig. 2E, right panel, and Supplementary Fig.  2 A). These proteins play roles in cytoskeleton and cell polarity regulation (ARHGEF17 and AMOTL1), metabolism and mitochondrial function (QDPR, NDUFV3) and gene regulation and chromatin remodeling (ZNF503, PHC2, PPME1). At the astrocyte stage, 131 DEPs were identified, with the number of up-regulated proteins four times higher than the number of down-regulated proteins (Fig. 2F, right panel, and Supplementary Fig.  2 A). These proteins include ion channels and transporters, cytoskeletal proteins and proteins regulating a plethora of biological processes, such as RNA binding and processing, transcription, metabolism and mitochondrial function, protein modification and degradation, and cell adhesion and signaling. Interestingly, the most significant DEP in astrocytes was TTYH3 (Tweety family member 3, adjusted p-value = 0.0045), which showed a Log_2_ fold change (Log_2_FC) increase of 1.6 in patient-derived astrocytes. The genotype difference observed in TTYH3 expression seems to be regulated at protein and not at mRNA level (Supplementary Fig. 2B). Interestingly, upregulation of the protein TTYH3, a calcium-activated chloride channel, was also recently reported in homozygous *Cln3*^∆ex7/8/∆ex7/8^ microglia [41], strengthening the hypothesis of a connection between CLN3 loss and TTYH3 induction.

By comparing transcriptome and proteome analyses, 849 and 766 common genes were identified in control and patient cells respectively, throughout development from iPSC to astrocyte (Supplementary Fig.  2 C). We observed a positive correlation between mRNA and protein changes during astrocyte differentiation in both control and patient comparisons (Supplementary Fig. 2D). While no significant correlation between DEGs and DEPs was observed in iPSC stage, three genes stood out, being upregulated in both proteome and transcriptome analyses at the astrocyte stage: HCFC1, PROM1, and CD70 (Supplementary Fig.  2 C). HCFC1 plays a role in chromatin organization and positive regulation of gene expression. CD70 plays a crucial role in immune system regulation. Interestingly, PROM1, a transmembrane glycoprotein that maintains stem cell properties, is implicated in retinal diseases.

### CLN3 loss leads to minor alterations in lysosomal processes in young astrocytes

**Fig. 3:**
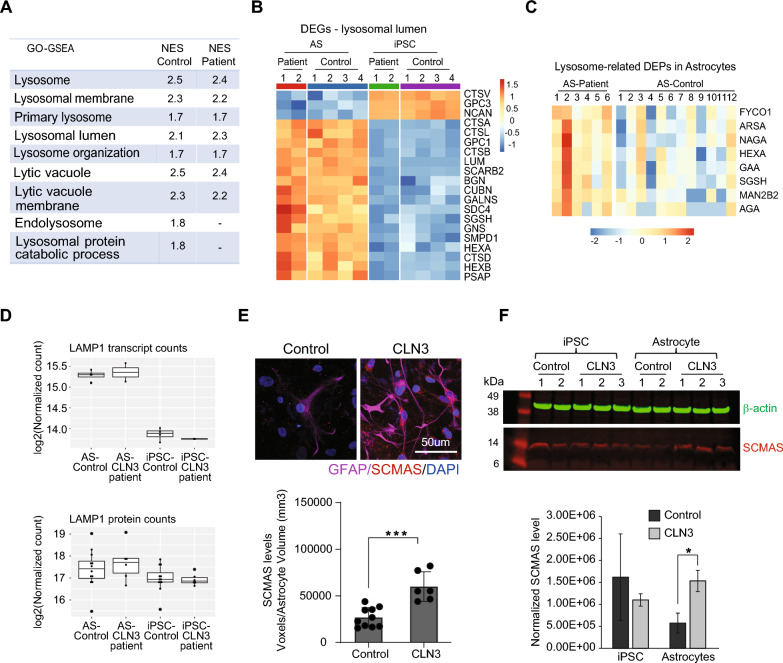
Gene and protein expression analyses reveal subtle dysregulation of lysosomal function in CLN3 patient-derived astrocytes. **A** Gene Set Enrichment Analysis (GSEA) of proteomic data shows similar Normalized Enrichment Score (NES) values for Gene Ontology (GO) terms associated with lysosomal function and homeostasis in control and patient cells throughout differentiation. **B** Expression profile of genes associated with the lysosomal lumen. For the control group, data from four samples (two clones from two control individuals) is presented. For the patient group, data from two clones of a single patient is presented. Each clone was collected in three independent differentiation experiments and pooled. **C** Levels of a subset of lysosomal proteins involved in the degradation of glycoconjugates in control and CLN3 patient-derived astrocytes. For the control group, data from four clones, two from each of two distinct control samples, and three differentiation experiments are presented. For the patient group, data from two clones and three differentiation experiments are presented. **D** Comparison of LAMP1 transcript and protein levels in iPSC and astrocyte stages across both genotypes. Student t-test revealed no significant differences between genotype comparisons. **E** Confocal analysis of subunit C of mitochondrial F1Fo ATP synthase (SCMAS, red) in control and patient-derived astrocytes, co-stained with GFAP (magenta). Nuclei were stained with DAPI. Quantification of SCMAS levels in astrocytes is shown in the lower panel. (***) indicates p-value < 0.001 based on student t-test. **F** Western-blot analysis of SCMAS (red) in both genotypes at the iPSC and astrocyte stages. Data from two control clones and three patient clones is shown. SCMAS levels were normalized to β-actin (green), with quantification shown in the lower panel. (*) indicates p-value < 0.05 based on t-test

The molecular features of CLN3 Batten disease are generally linked to lysosomal dysfunction. Interestingly, only small alterations in the levels of proteins associated with lysosomal processes were identified in CLN3 patient-derived cells compared to control (Supplementary Fig. 3). Gene Ontology-based Gene Set Enrichment Analysis (GO-GSEA) of proteome data from iPSC to astrocytic stage revealed similar positive Normalized Enrichment Score (NES) values for enriched GO terms related to lysosome for both genotypes (Fig. 3A). This data indicates that most of the biological processes represented by these GO-terms are upregulated at similar extent in both control and CLN3 patient groups during the transition from iPSC to astrocytic stage. Notably, upregulation of “endolysosome” and “lysosomal protein catabolic process” was identified only in the control group and not significantly active or enriched in the patient samples, suggesting that these processes might be partly disrupted or less active in the patient samples. At transcript level, we found similar regulations of genes involved in lysosomal lumen in both genotypes (Fig. 3B). A subset of genes, however, appear to exhibit greater downregulation in iPSCs and more pronounced upregulation in astrocytes derived from the CLN3 patient compared to the control (e.g., *SDC4*, *SGSH*, *GNS*, *SMPD1*, *HEXA*, *CTSD* and *HEXB*). These genes regulate the degradation of glycoconjugates (SGSH, GNS, HEXA and HEXB), cell–matrix interactions (*SDC4*) and the conversion of sphingomyelin into ceramide within lysosomes (*SMPD1*). At protein level, we found upregulation of a subset of lysosomal proteins involved in the breakdown of glycogen, glycoconjugates, oligosaccharides, and sugar moieties (Fig. 3C). Among them, the upregulation of HEXA and SGSH was also found in the transcriptome data (Fig. 3B). The levels of LAMP1, a key lysosomal marker, are increased in astrocytes compared to iPSCs both at mRNA and at protein levels. The differences between the two genotypes in these comparisons, however, are not significant, suggesting a similar number of lysosomes in both control and patient cells (Fig. 3D).

To further assess lysosomal regulation, we investigated the levels of subunit C of mitochondrial F1Fo ATP synthase (SCMAS) in patient-derived cells. Our data show a significant increase in SCMAS accumulation in CLN3 patient-derived astrocytes compared to controls (Fig. 3E and F). No difference in SCMAS levels was observed between patient and control at the iPSC stage. Furthermore, CLN3 astrocytes did not exhibit detectable autofluorescent storage material associated with Batten disease, most likely due to limited culture duration.

### CLN3 deficiency leads to marked mitochondrial dysregulation

**Fig. 4:**
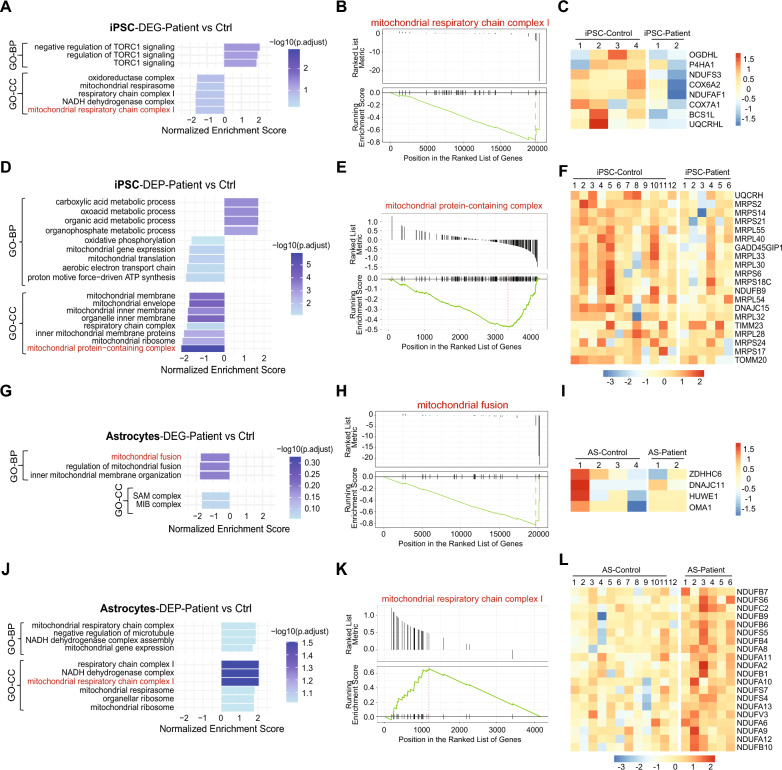
Gene Ontology (GO) Gene Set Enrichment Analysis (GSEA) reveals enrichment of mitochondria-associated terms in CLN3 patient-derived iPSC and astrocytes. (**A**, **D**) Two-sided bar plots of GSEA results from (**A**) gene and (**D**) protein expression data showing normalized enrichment scores (NES) for top GO terms, categorized by Biological Process (BP) and Cellular Component (CC), comparing CLN3 patient-derived iPSCs to controls. (**B**, **E**) Gene set enrichment plot showing downregulation of genes (**B**) and (**E**) proteins for pathways highlighted in red in panels **A** and **D**, respectively. (**C**, **F**) Heatmaps of selected (**C**) genes and (F) proteins corresponding to pathways highlighted in red in panels A/B and D/E, respectively. (**G**, **J**) Two-sided bar plots of top pathways identified by GSEA of (**G**) gene and (**J**) protein expression data comparing CLN3 patient-derived astrocytes to controls. (**H**, **K**) Enrichment plots for pathways highlighted in red in panels G and **J**, based on (**H**) gene and (**K**) protein analyses. (**I**, **L**) Heatmaps of selected (**I**) genes and (**L**) proteins associated with the pathways highlighted in red in the respective comparisons

Our omics data revealed that the most pronounced alterations in transcript and protein levels due to CLN3 loss were related to mitochondrial homeostasis and function. In iPSC, GO-GSEA of gene expression data revealed underrepresentation of terms related to the mitochondrial respiratory chain complex I in CLN3 patient-derived cells (Fig. 4A–C). Downregulation of mitochondrial respirasome could be linked to negative regulation of the Target of Rapamycin Complex 1 (TORC1), a crucial regulator of cell growth and metabolism (Fig. 4A). At protein level, negative NES values were identified for GO terms associated with expression and translation of mitochondrial genes, mitochondrial membranes, cellular respiration and oxidative phosphorylation (Fig. 4D)**.**

Underrepresentation of mitochondrial protein-containing complex and levels of genes involved in this process are illustrated in Fig. 4E and F, respectively. Conversely, terms associated with small molecule metabolism, fatty acid metabolism and processing of breakdown products of fats, carbohydrates, and/or proteins (organic/carboxylic acids, small molecules) were overrepresented in the CLN3 patient group (Fig. 4D).

In CLN3 astrocytes, GO-GSEA of the transcriptome data identified underrepresentation of mitochondrial fusion and inner mitochondrial membrane organization (Fig. 4G–I). Interestingly, we found the opposite representation of terms related to mitochondrial respiration compared to iPSC. GO-GSEA of protein profiles revealed overrepresentation of mitochondrial gene expression, NADH dehydrogenase, electron transfer activities and mitochondrial respirasome, particularly, the respiratory chain complexes I and IV, in CLN3 astrocytes (Fig. 4J and supplementary Fig.  4 A). Overrepresentation of the mitochondrial respiratory complex I and the expression level of key proteins involved in this process are illustrated in Fig. 4K and L, respectively.

To determine whether the elevated levels of mitochondrial respiratory supercomplex proteins in CLN3 astrocytes were due to an increased number of complexes within the inner mitochondrial membranes or a higher number of mitochondria, we examined the mitochondrial DNA copy number in astrocytes derived from control and the CLN3 patient (Supplementary Fig. 4B). This data indicates a similar number of mitochondria in both control and patient astrocytes, suggesting an increased number of respiratory supercomplex proteins within the mitochondrial membrane. This observation agrees with potential defects in inner mitochondrial membrane organization illustrated in Fig. 4G.

### CLN3 deficiency in iPSC-derived astrocytes leads to a shift in fatty acid metabolism

**Fig. 5:**
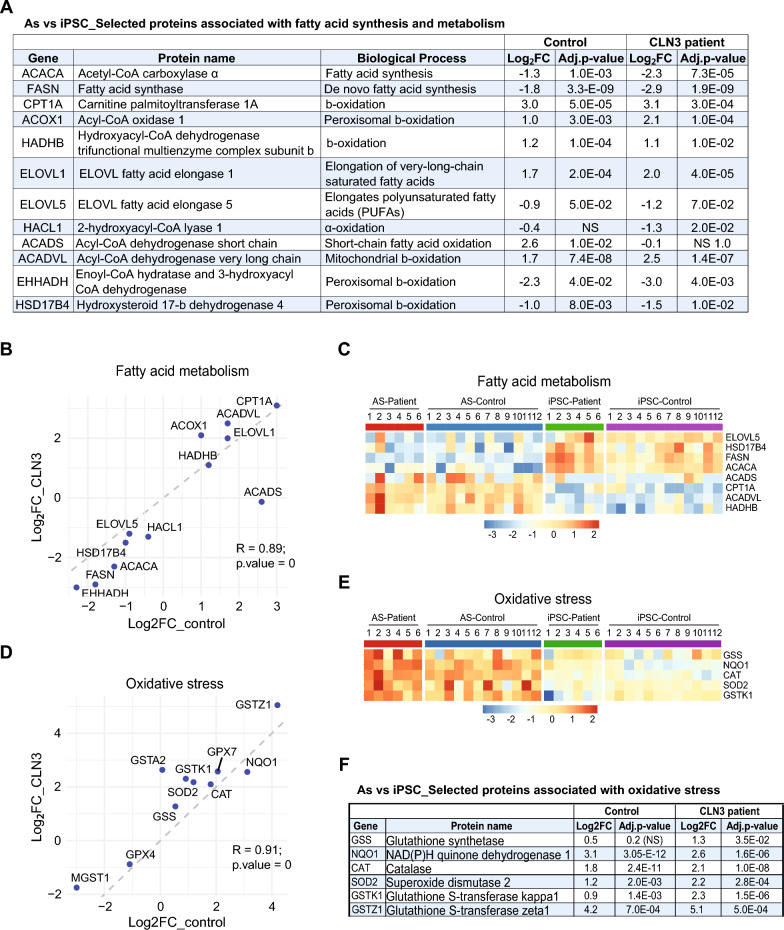
Proteome analysis reveals a shift in fatty acid metabolism and enhanced oxidative stress response in CLN3 patient-derived cells. **A** Changes in levels of selected proteins involved in fatty acid synthesis and metabolism during the transition from iPSC to astrocyte stages in CLN3 patient and control groups. Log_ fold changes and adjusted p-values are indicated. **B** Correlation plot showing the protein expression changes (log₂ fold change, astrocyte vs. iPSC) for control cells (x-axis) and CLN3 patient cells (y-axis) for the fatty-acid-metabolism proteins listed in **A**. Each point represents how a protein is regulated during the iPSC-to-astrocyte transition in each group. **C** Heatmap of a subset of the proteins described in **A** and **B**. **D** Correlation plot showing the protein expression changes (log₂ fold change, astrocyte vs. iPSC) for control cells (x-axis) and CLN3 patient cells (y-axis) for selected proteins involved in intracellular oxidative-stress responses. Each point represents the differentiation-associated change in that protein between stages. **E** Heatmap of a subset of the proteins shown in **D**. **F** Changes in levels of selected proteins involved in oxidative stress responses during the transition from iPSC to astrocyte stages in CLN3 patient and control groups. Log_ fold changes and adjusted p-values are indicated

Lipid metabolism is dependent on mitochondria function and because mitochondria dynamics is differentially regulated in CLN3 astrocytes, we also examined the levels of proteins regulating fatty acid metabolism in our datasets. The level of ELOVL1, an enzyme responsible for the elongation of very-long-chain saturated fatty acids, is increased in the patient cells, indicating a shift towards the production of longer-chain saturated fatty acids (Fig. 5A and B). In addition, a minor decrease in ELOVL5 levels was observed in the patient-derived cells, suggesting a reduction in the synthesis of polyunsaturated fatty acids (PUFAs), which are crucial for maintaining membrane fluidity and signaling functions (Fig. 5A–C).

Interestingly, ACOX1 and ACADVL, the enzymes that catalyze initial steps of β-oxidation of very long-chain fatty acids in the peroxisomes and long-chain fatty acids in mitochondria, respectively, are upregulated in the CLN3 group (Fig. 5A and B). In addition, we found downregulation of enzymes involved in fatty acid synthesis (e.g. ACACA, FASN) and oxidation (HACL1, EHHADH, HSD17B4). Reduced levels of ACACA and FASN may indicate a shift away from lipid synthesis towards increased fatty acid oxidation. Moreover, ACADS is upregulated from iPSC to astrocyte stage only in healthy cells, supporting efficient energy production through enhanced mitochondrial β-oxidation of short-chain fatty acid during differentiation. In contrast, the absence of significant ACADS upregulation in the patient cells suggests a disrupted metabolic adaptation during differentiation. Interestingly, the changes in levels of CPT1A and HADHB are similar in both genotypes throughout differentiation. CPT1A is essential for the transport of long-chain fatty acids into mitochondria for β-oxidation, while HADHB is involved in the final steps of β-oxidation. This data suggests that some aspects of fatty acid transport and β-oxidation remain stable, possibly maintaining a baseline level of energy production.

### The levels of key oxidative stress response proteins are increased in CLN3 astrocytes

Changes in mitochondrial function and lipid metabolism play a key role in triggering intracellular oxidative stress response. Thus, we investigated the expression pattern of proteins involved in this process in these cells (Fig. 5D–F). We found increased levels of a subset of antioxidant proteins in the CLN3 group from iPSC to astrocyte stage. For instance, the levels of Glutathione synthetase (GSS) and Glutathione S-transferase kappa 1 (GSTK1) were substantially increased in the CLN3 group compared to control throughout differentiation. In addition, a modest increase in Glutathione S-Transferase Zeta 1 (GSTZ1) was also observed in CLN3 deficient cells. GSS catalyzes the production of glutathione, a key intracellular ROS neutralizer, while GSTK1 and GSTZ1 promote cellular detoxification by catalyzing the conjugation of glutathione to various hydrophobic substances or electrophilic compounds, respectively, aiding their removal from cells. Interestingly, while the basal levels of GSS appear to be lower in control iPSC, the basal levels of GSTK1 are considerably lower in the patient iPSC. Similarly, lower basal levels of the antioxidant proteins Catalase (CAT) and NAD(P)H Quinone Dehydrogenase 1 (NQO1) are observed in the control iPSC compared to patient iPSC. The levels of these proteins, along with the antioxidant enzyme SOD2 (mitochondrial superoxide dismutase), are markedly elevated in the CLN3 astrocytes compared to controls (Fig. 5D–F).

### Extracellular matrix dynamics are affected by CLN3 loss

Our transcriptome data revealed dysregulation of a subset of ECM genes in the CLN3 astrocytes (Supplementary Fig.  5 A), indicating that these cells are undergoing a complex reactive transformation, characterized by ECM remodeling (MMP2, PCOLCE, LAMC2, ADAMTS19, ASPN, OGN, MFAP4, ANGPT2 and collagens COL6A3, COL12A1, COL28A1, COL2A1), cell adhesion, migration and astrocyte-ECM interactions (LOXL2, LAMC2), altered synaptic support and plasticity (WNT7A, LRRTM1, RELN, AGRN), neuroinflammatory signaling (TRIL), reduced regenerative capacity (GDF10, FGFR2) and decreased support to neuron integrity and survival (NDNF, COL2A1 and GDF10).

However, the proteome data (Supplementary Fig. 5B) does not reflect these transcriptome changes. Only a subset of ECM-associated proteins was detected, and these did not show the same regulatory patterns observed at the mRNA level. This discrepancy suggests that the dysregulation of ECM-related genes at the transcript level may involve non-coding or regulatory functions of RNA.

### CLN3 deficiency impacts chromatin organization and remodeling

**Fig. 6:**
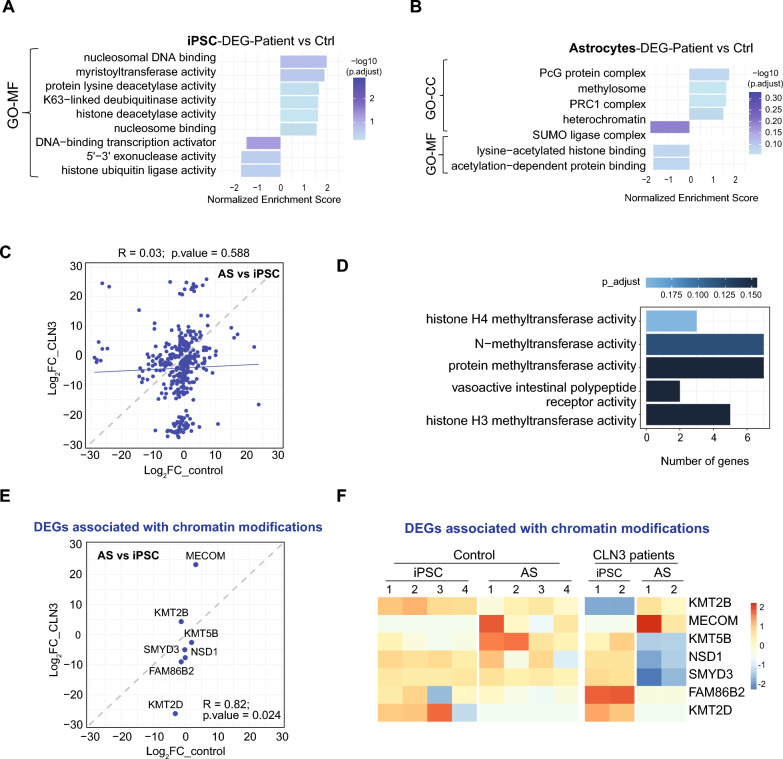
CLN3 deficiency alters chromatin-associated gene expression during astrocyte differentiation. **A**, **B** gene expression data showing normalized enrichment scores (NES) for top GO terms, categorized by Molecular Function (MF) and Cellular Component (CC) in **A** iPSC and **B** astrocytes, comparing CLN3 patient-derived cells to controls. **C** Correlation plot showing Log_ fold change values for genes identified by multifactorial analysis. **D** Overrepresentation of molecular function terms related to histone methylation and chromatin remodeling. **E** Correlation plot showing Logfold change values for selected genes associated with chromatin modifications. **F** Heatmap of the subset of the genes shown in **E**

The comparison between the transcriptome profiles of patient and control iPSC revealed a negative regulation of protein-DNA binding activities associated with transcription activation (i.e., DNA-binding transcription activator, 5’−3’ exonuclease activities) and chromatin remodeling (i.e., histone ubiquitin ligase activity) (Fig. 6A). In addition, positive regulation of terms related to deacetylase activities suggest a shift toward tighter chromatin and reduced transcriptional plasticity. We also found altered signaling and membrane dynamics (via myristoylation) which could impact neural development and synaptic function, as well as changes in the fine-tuned control of DNA–protein interactions via ubiquitin signaling.

At astrocyte stage, there is a negative regulation of terms reflecting a reduction in the recruitment of protein complexes to chromatin (regulation of protein localization to chromatin), recognition of acetylated histones typically associated with open chromatin and active transcription (lysine acetylated histone binding, acetylation-dependent protein binding), and fine-tuning transcriptional repression (SUMO ligase complex), potentially leading to impaired gene activation and aberrant chromatin states. Moreover, we found upregulation of terms associated with epigenetic (PcG protein complex, PRC1 complex) and gene expression (methylosome, heretochromatin) repression (Fig. 6B). This data suggests global epigenetic reprogramming toward a more repressive, compacted chromatin landscape in the patient cells.

To further explore the molecular networks most affected by CLN3 deficiency, a multifactorial transcriptomic analysis was conducted to disentangle the individual and interactive effects of genotype (control vs. CLN3 patient) and cell type (iPSC vs. astrocyte) on gene expression (Supplementary Table 1E). This analysis revealed 363 genes with statistically different patterns of expression in CLN3 patient in the trajectory of astrocyte differentiation (AS vs iPSC) considering p.adj < 0.05 and Log_2_FC ≥ 1. A correlation plot of log₂ fold change values for genes identified as DEGs in the multifactorial analysis is illustrated in Fig. 6C. Overrepresentation of these genes in molecular function highlighted terms related to chromatin modification, particularly, DEGs involved in histones H3 and H4 lysine methylation activities, suggesting that CLN3 deficiency may impact chromatin remodeling and organization (Fig. 6D). DEGs associated with chromatin modifications are displayed in the correlation plot (Fig. 6E) and heat map (Fig. 6F). Notably, the expression of the subset of chromatin-modifying genes shown in Fig. 6D remains unchanged or shows mild alterations in the Control group during the transition from iPSCs to astrocytes. In contrast, these genes exhibit marked differential regulation in CLN3 patient samples, suggesting that the absence of CLN3 leads to significant epigenetic alterations during astrocyte differentiation.

## Discussion

Progressive intracellular accumulation of SCMAS, starting within the lysosomes, is a hallmark of CLN3 Batten disease [42]. The storage accumulation is most prominent in neurons and has also been reported in microglia [5, 41, 43]. Although these intracellular inclusions are not considered to be a direct cause of the neuropathology characteristic of NCL diseases [6, 44], it is used as indicators of disease progression [40]. Our data indicate that, in young astrocytes, CLN3 loss has a minor impact on lysosomal homeostasis. We observed, however, upregulation of proteins involved in glycoconjugate catabolism, which may represent an attempt to enhance the clearance of these compounds, potentially compensating for early lysosomal pathology in these cells. The small, although significant, increase in SCMAS levels in patient astrocytes indicates initial signs of defective or overwhelmed lysosomal degradation pathways. If this hypothesis is accurate, SCMAS accumulation, lysosomal pathway dysregulation, and the presence of autofluorescent storage material, are likely to be more pronounced in astrocytes cultured for extended periods.

Abnormal mitochondrial activity, alterations in translation and decreased protein synthesis due to *CLN3* loss have also been reported in Hela cells [45]. Reduced mitochondrial respiration and ATP production has been reported in *Cln3*^Δex7/8^ murine astrocytes, both under resting conditions and upon exposure to proinflammatory cytokines [46]. Moreover, mRNA profiling of the eyes of *Cln3*-knockout mice showed downregulation of genes associated with energy production as early as in 10-weeks old mice compared to WT mice [47]. Interestingly, a link between downregulation of mitochondrial respirasome and negative regulation of TORC1 was reported in fission yeast *Schizosaccharomyces pombe* lacking the *btn1* gene, a homologue of human *CLN3*  [48]. These reports indicate a downregulation of mitochondrial oxidative respiration, and a possible association to TORC1 repression, as we observed in CLN3 iPSC.

Intriguingly, our data supports an increase in the levels of mitochondrial respiratory supercomplex proteins in CLN3 astrocytes, perhaps leading to abnormalities in the inner mitochondrial membrane, which could contribute to increased mitochondrial respiration and ATP production. This contrasts with the findings in *cln3* murine astrocytes and non-neural *CLN3* human cells, suggesting that CLN3 may have additional or distinct roles in human astrocytes compared to these models.

Approximately 20% of the brain’s total energy expenditure is attributed to the oxidation of free fatty acids—a process that occurs primarily in astrocytes [49, 50]. Importantly, a reduced rate of mitochondrial fatty acid synthesis has been reported in CLN3 patient fibroblasts, associated with defects in mitochondrial membrane integrity [51]. In this study, we found a significant shift in fatty acid metabolism in CLN3 patient-derived cells, suggesting metabolic adaptations in response to mitochondrial dysfunction. Fatty acid breakdown proceeds via two main pathways: α-oxidation, which primarily occurs in peroxisomes and processes branched-chain fatty acids, and β-oxidation, which begins with the breakdown of very long-chain fatty acids (VLCFA) in peroxisomes. The resulting shorter-chain fatty acids are subsequently transported to mitochondria for further oxidation and ATP production [52]. We found elevated levels of ELOVL1 in patient-derived cells, which may enhance the synthesis of longer-chain saturated fatty acids. Notably, elevated ELOVL1 activity has been linked to neurotoxicity in astrocytes, potentially contributing to cellular stress and damage [53, 54]. To possibly meet the increased demand for VLCFA, patient cells also showed higher expression of ACOX1 and ACADVL. While elevated ACADVL levels may reflect an attempt to enhance fatty acid oxidation in response to mitochondrial dysfunction, the upregulation of ACOX1 points to a compensatory mechanism to manage fatty acid metabolism due to impaired mitochondrial β-oxidation. In addition, the downregulation of HACL1, involved in α-oxidation of branched-chain fatty acids, may reflect altered fatty acid metabolism pathways in response to mitochondrial stress. Moreover, downregulation of EHHADH and HSD17B4, both involved in peroxisomal β-oxidation, suggest changes in peroxisomal function due to mitochondrial dysfunction, potentially leading to VLCFA accumulation. Despite the increased levels of proteins from respiratory supercomplexes I and IV, CLN3 deficient cells may struggle to effectively upregulate ACADS during differentiation, potentially leading to impaired short-chain fatty acid oxidation.

Astrocytes are important ROS handlers in the brain. They have higher tolerance to neurotoxic molecules and harbor higher levels of antioxidant systems compared to neurons and can protect neurons from oxidative stress [55–63]. Our data suggest that CLN3 deficiency leads to enhanced activity of mitochondrial supercomplexes and abnormal lipid metabolism in astrocytes, which may increase ROS production in these cells. For example, ACOX1 upregulation can increase the rate of peroxisomal β-oxidation of very long-chain fatty acids, while producing hydrogen peroxide [64, 65]. Moreover, the downregulation of the following enzymes in this pathway could cause the accumulation of partially oxidized fatty acids and toxic intermediates. Hence, the increased ROS production from ACOX1 activity combined with the metabolic imbalance caused by impaired detoxification could lead to significant oxidative stress and cellular damage, also contributing to inflammatory responses.

In iPSC, we observed lower basal levels of GSS in the control group, while the GSTK1 basal levels were lower in the patient iPSC. This data suggests that, at iPSC stage, an increase in glutathione production was possibly required in patient iPSC to handle potential increased basal ROS levels in these cells, while, throughout differentiation, a stronger requirement for glutathione detoxification became eminent, leading to marked upregulating the GSTK1 levels in the patient cells. At astrocytic stage, the level of several proteins involved in antioxidative response is evident in the patient group, indicating an adaptive anti-ROS response, perhaps to cope with increased ROS levels generated by dysfunctional fatty acid metabolism and up-regulated oxidative phosphorylation. Increased ROS levels have been reported in CLN3 lymphoblasts and fibroblasts [66, 67]. In *Drosophila*, loss of CLN3 was correlated to increased oxidative stress, whereas CLN3 overexpression conferred resistance against oxidative stress. These reports and our findings indicate that CLN3 plays a role in oxidative stress response and connects this function to neuronal degeneration [68].

A limitation of this study is that all CLN3 patient‑derived replicates originate from a single individual; therefore, we cannot fully exclude that some observed phenotypes may reflect patient‑specific genetic background effects rather than solely the loss of functional CLN3. Future studies including additional patient lines or isogenic controls would further strengthen these findings.

## Conclusions

Mitochondrial dysfunction is implicated in several neurodegenerative disorders, such as Alzheimer’s disease (AD), Parkinson’s disease (PD), Huntington’s disease (HD), and amyotrophic lateral sclerosis (ALS) [69]. A common link of different neurodegenerative disorders appears to be the accumulation of the misfolded proteins, which interact and disturb the electron transport chain and other mitochondrial functions. This disruption leads to alteration in lipid metabolism, energy depletion, increased ROS production, activation of inflammation and impaired mitophagy [70, 71]. However, whether the misfolding of proteins is the cause or a consequence of mitochondrial dysfunction remains unclear. Hypothetically, restoring mitochondrial function in CLN3 astrocytes could improve neuron health in CLN3 patients, possibly delaying disease progression. Further investigation and functional validation of the biological networks identified in this study using human preclinical models of higher complexity, such as brain organoid models, can shed light into the temporal relationship between mitochondrial dysfunction in astrocytes and neuronal death, as well as well as the broader implications for other neuronal cell types in CLN3 Batten disease. Insights into disease mechanisms can lead to the identification of therapeutic targets, accelerating the development of new therapies. Importantly, better understanding of the mechanism of neurodegeneration in rare neurological disorders, such as neuronal ceroid-lipofuscinoses, can enhance our knowledge of the mechanisms involved in more complex genetic neurodegenerative conditions, including those associated with aging.

## Supplementary Information

Additional file1** Supplementary Fig. 1. **Characterization of iPSC derived from Control and CLN3 patient fibroblasts. A Representative brightfield images of iPSC cell morphology, acquired at 10 × magnification. Scale bars: 800 μm. B mRNA levels of the pluripotency markers NANGO, OCT4 and SOX2 assessed via qPCR. For validation, gene expression levels of pluripotent markers in iPSC were compared to levels in fibroblasts. C Immunocytochemistryanalysis of pluripotency markers showing expression of NANOG, OCT4 and TRA160 proteins in the iPSC. Scale bars: 200 μm. D Confirmation of mesodermal and endodermal differentiation of iPSCs via IHC analysis of the mesodermal markers Brachyuryand CXCR4, and endodermal markers SOX17and CXCR4. Nuclei are stained by DAPI; Scale bars: 200 μm. E Confirmation of CLN3 genotype in patient cells. Illustration of the mutation analysis strategy for confirmation of CLN3 genotype described by Järvelä et al.. Primer combination 1 amplifies both normal and mutated CLN3, while primer combination 2 amplifies only the normal CLN3 gene, as one of the primers anneal within the deleted sequences in the 1 kb CLN3 mutation. Gel electrophoresis of PCR products as confirming the presence of two mutated CLN3 alleles in the patient-derived iPSCs, and presence of two normal CLN3 alleles in the healthy control-derived iPSC. **Supplementary Fig. 2. **Proteomic changes during differentiation and correlation with transcriptomic profiles. A Volcano plot analysis of proteome data comparing control and CLN3 patient cells during differentiation from iPSC to astrocytes, as well as stage specific comparisons within each genotype. Differentially expressed proteinsare highlighted as back dots in all comparisons. B Box plot showing transcript and protein levels of TTYH3, the most significantly altered DEP in patient astrocytes compared to control. (**) indicates adjusted p-value < 0.01. C Venn diagrams illustrating the number of unique and overlapping genes identified in transcriptomic and proteomic datasets for both differentiation and stage-specific comparisons. D Correlation plots showing a positive correlation between transcriptomic and proteomic data during differentiation. No significant correlation was observed in stage-specific comparisons, likely due to the limited number of overlapping genes in these datasets. **Supplementary Fig. 3. **Heatmap of proteins involved in lysosomal function in CLN3 patient and control groups at distinct differentiation stages. The number of biological replicates for each group is indicated. For the control group, data from four clones, two from each of two distinct control samples, and three differentiation experiments are presented. For the patient group, data from two clones and three differentiation experiments are presented. **Supplementary Fig. 4. **Assessment of mitochondrial complex IV protein levels and mitochondrial DNAcopy number in CLN3 patient-derived cells. A Heatmap of proteins belonging to the mitochondrial respiratory chain supercomplex IV in CLN3 patient and control groups at iPSC and astrocyte stages. B Relative mtDNA copy number was assessed in astrocytes using quantitative PCR. Total DNA was extracted, and mtDNA levels were quantified by amplifying the mitochondrial-encoded gene RNR1, normalized to the nuclear-encoded reference gene HBB. Data are presented as fold change relative to the nuclear gene for CLN3 patient and control astrocytes. Error bars represent the standard deviationrepresenting the mean of three technical replicates from two independent biological experiments. Statistical analysis using the student t-test revealed no significant differences between the two groups. **Supplementary Fig. 5. **Heatmap of genes involved in extracellular matrix organization in CLN3 patient and control groups at distinct differentiation stages. A mRNA expression levels and B protein abundance of ECM-associated genes. The number of replicates for each group is indicated, as described in Fig. 3. **Supplementary Fig. 6. **Uncropped Western blot images shown in Fig. 3F. Blots were probed for SCMAS with β-actin included to confirm equal protein loading across lanes. Molecular weight markers are indicated, and all lanes correspond to patient-derived and control astrocyte samples as described in the main figure. No additional modifications were made to the blot images beyond cropping for presentation in the main figure.

Additional file2** Supplementary table S1.**

Additional file3** Supplementary table S2.**

## Data Availability

The original uncropped version of western-blot analysis shown in Fig. 3F is provided in Supplementary Fig. 6. Raw data files are available from the corresponding author(s) upon reasonable request and subject to institutional and ethical approvals. The LFQ MS data can be downloaded from the ProteomeXchange Consortium via the PRIDE partner repository with the dataset identifier PXD064202 following Reviewer account details: Username: reviewer_pxd064202@ebi.ac.uk. Password: NxhB5U9jR14F.
