# Supplementary material for: Modeling CLN3 Batten disease in astrocytes reveals alterations in mitochondria homeostasis, fatty acid metabolism and oxidative stress response
Source: J Biomed Sci. 2026 May 13;33:50. doi: 10.1186/s12929-026-01253-y (PMC13173811; doi:10.1186/s12929-026-01253-y)
Supplement: Supplementary file 1 — Additional file1 Supplementary Fig. 1. Characterization of iPSC derived from Control and CLN3 patient fibroblasts. A Representative brightfield images of iPSC cell morphology, acquired at 10 × magnification. Scale bars: 800 μm. B mRNA levels of the pluripotency markers NANGO, OCT4 and SOX2 assessed via qPCR. For validation, gene expression levels of pluripotent markers in iPSC were compared to levels in fibroblasts. C Immunocytochemistryanalysis of pluripotency markers showing expression of NANOG, OCT4 and TRA160 proteins in the iPSC. Scale bars: 200 μm. D Confirmation of mesodermal and endodermal differentiation of iPSCs via IHC analysis of the mesodermal markers Brachyuryand CXCR4, and endodermal markers SOX17and CXCR4. Nuclei are stained by DAPI; Scale bars: 200 μm. E Confirmation of CLN3 genotype in patient cells. Illustration of the mutation analysis strategy for confirmation of CLN3 genotype described by Järvelä et al.. Primer combination 1 amplifies both normal and mutated CLN3, while primer combination 2 amplifies only the normal CLN3 gene, as one of the primers anneal within the deleted sequences in the 1 kb CLN3 mutation. Gel electrophoresis of PCR products as confirming the presence of two mutated CLN3 alleles in the patient-derived iPSCs, and presence of two normal CLN3 alleles in the healthy control-derived iPSC. Supplementary Fig. 2. Proteomic changes during differentiation and correlation with transcriptomic profiles. A Volcano plot analysis of proteome data comparing control and CLN3 patient cells during differentiation from iPSC to astrocytes, as well as stage specific comparisons within each genotype. Differentially expressed proteinsare highlighted as back dots in all comparisons. B Box plot showing transcript and protein levels of TTYH3, the most significantly altered DEP in patient astrocytes compared to control. (**) indicates adjusted p-value < 0.01. C Venn diagrams illustrating the number of unique and overlapping genes identified in transcri [file 12929_2026_1253_MOESM1_ESM.pdf]

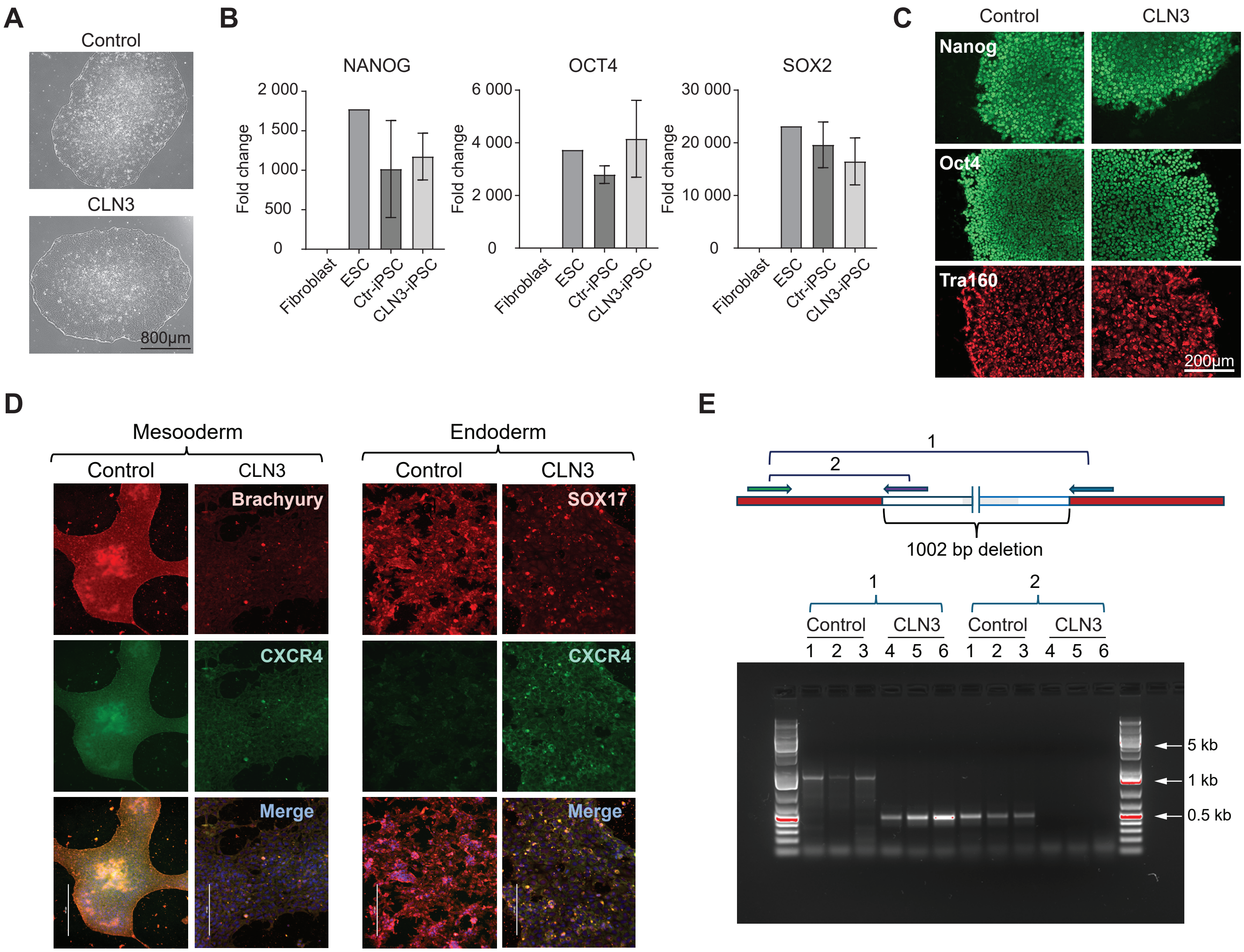

Supplementary figure 1. Characterization of iPSC derived from Control and CLN3 patient (CLN3) fibroblasts. (**A**) Representative brightfield images of iPSC cell morphology, acquired at 10x magnification. Scale bars: 800 µm (**B**) mRNA levels of the pluripotency markers NANGO, OCT4 and SOX2 assessed via qPCR. For validation, gene expression levels of pluripotent markers in iPSC were compared to levels in fibroblasts (FB). (**C**) Immunocytochemistry (ICC) analysis of pluripotency markers showing expression of NANOG, OCT4 and TRA160 proteins in the iPSC. Scale bars: 200 µm. (**D**) Confirmation of mesodermal and endodermal differentiation of iPSCs via IHC analysis of the mesodermal markers Brachyury (red) and CXCR4 (green), and endodermal markers SOX17 (red) and CXCR4 (green). Nuclei are stained by DAPI (blue); Scale bars: 200 µm. (**E**) Confirmation of CLN3 genotype in patient cells. Illustration of the mutation analysis strategy for confirmation of CLN3 genotype described by Järvelä et al. (1996). Primer combination 1 amplifies both normal and mutated CLN3, while primer combination 2 amplifies only the normal CLN3 gene, as one of the primers anneal within the deleted sequences in the 1kb CLN3 mutation. Gel electrophoresis of PCR products as confirming the presence of two mutated CLN3 alleles in the patient-derived iPSCs (CLN3), and presence of two normal CLN3 alleles in the healthy control-derived iPSC (Control).

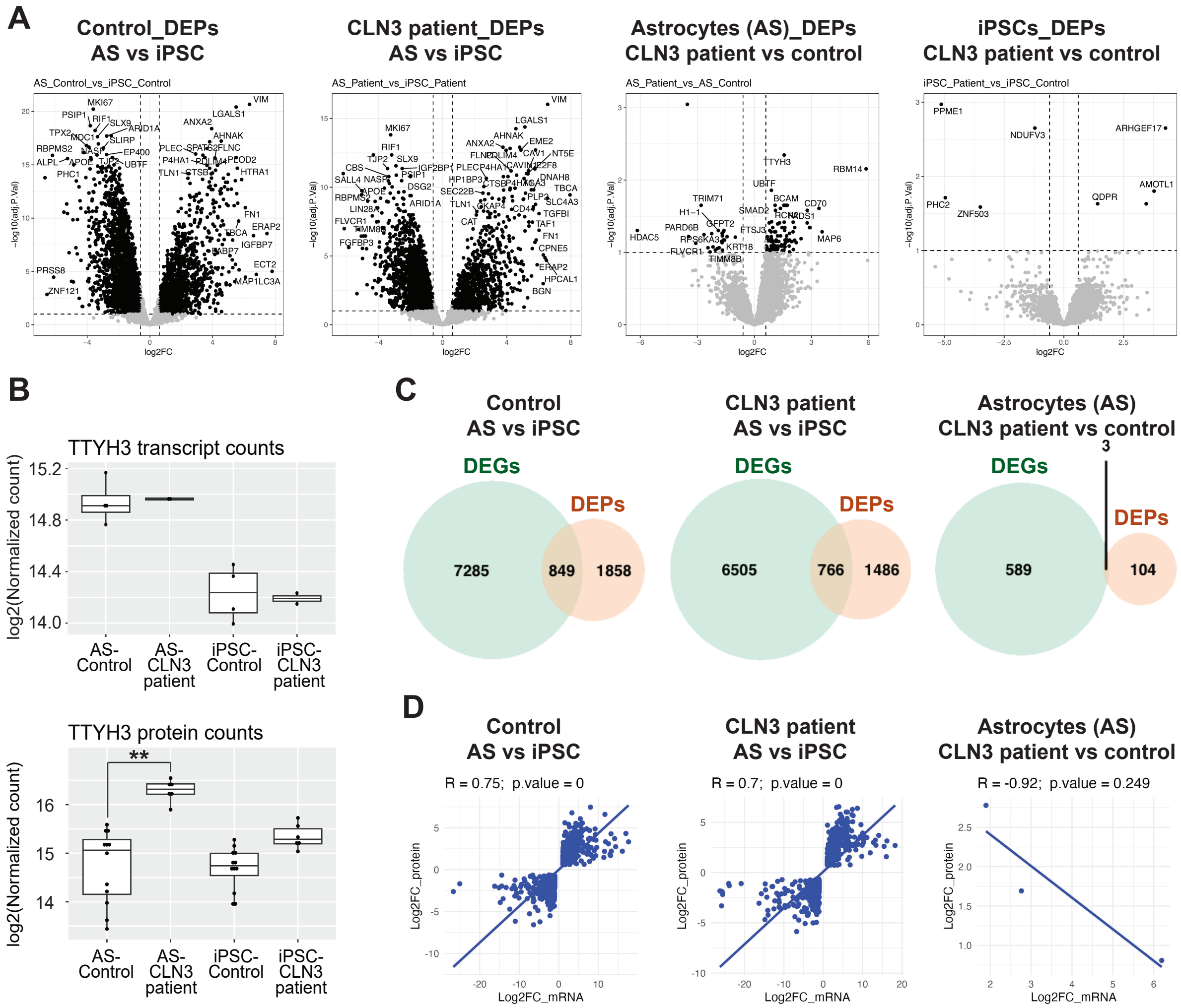

Supplementary figure 2. Proteomic changes during differentiation and correlation with transcriptomic profiles. (**A**) Volcano plot analysis of proteome data comparing control and CLN3 patient cells during differentiation from iPSC to astrocytes, as well as stage specific comparisons within each genotype. Differentially expressed proteins (DEPs) are highlighted as back dots in all comparisons. (**B**) Box plot showing transcript and protein levels of TTYH3, the most significantly altered DEP in patient astrocytes compared to control. (**) indicates adjusted p-value<0.01. (**C**) Venn diagrams illustrating the number of unique and overlapping genes identified in transcriptomic and proteomic datasets for both differentiation and stage-specific comparisons. (**D**) Correlation plots showing a positive correlation between transcriptomic and proteomic data during differentiation. No significant correlation was observed in stage-specific comparisons, likely due to the limited number of overlapping genes in these datasets.

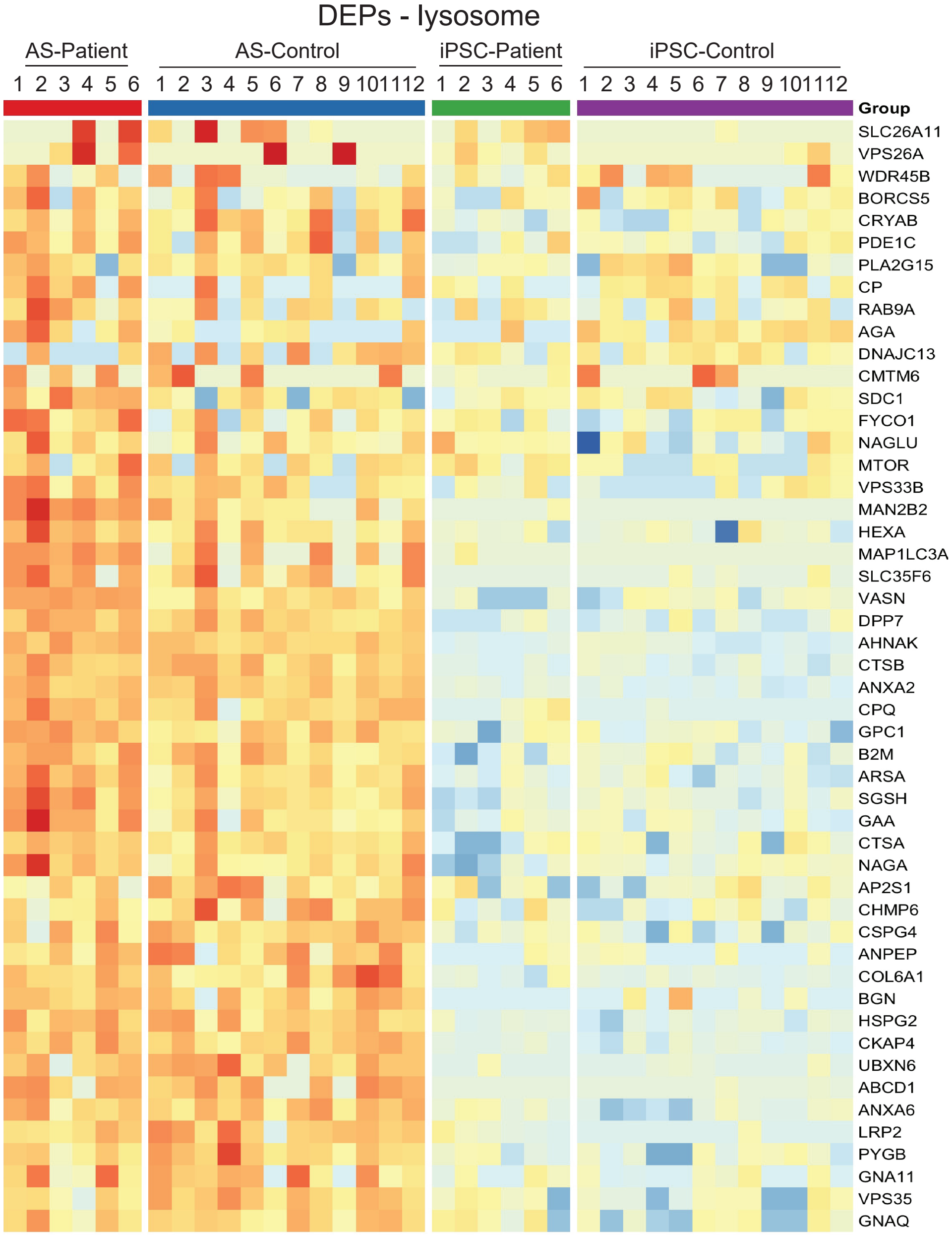

Supplementary figure 3. Heatmap of proteins involved in lysosomal function in CLN3 patient and control groups at distinct differentiation stages (iPSC and astrocyte). The number of biological replicates for each group is indicated. For the control group, data from four clones, two from each of two disctinct control samples, and three diferentiation experiments are presented. For the patient group, data from two clones and three diferentiation experiments are presented.

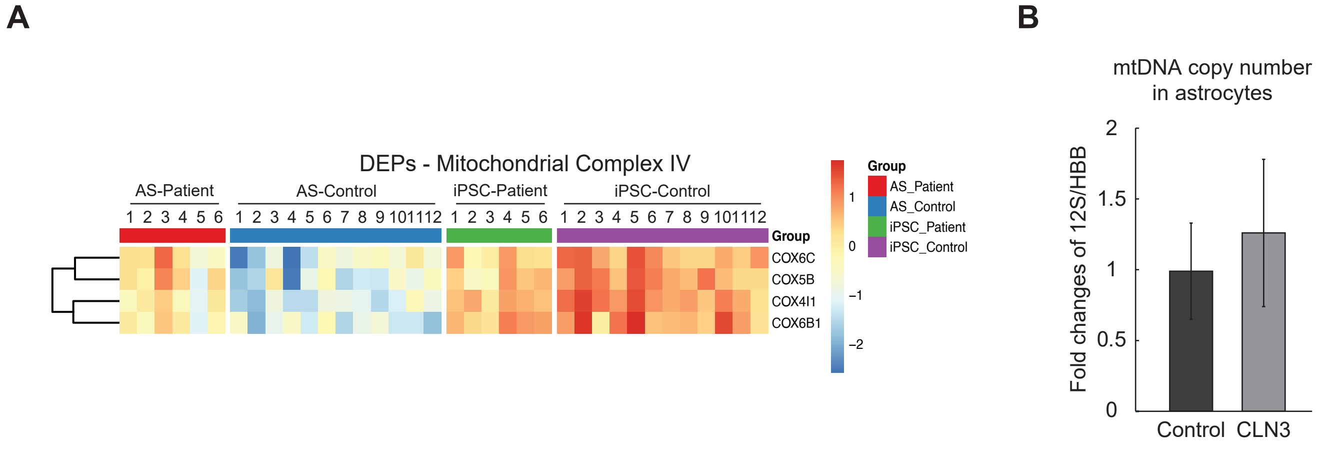

Astrocytes	AVE	SD
Crtl	0.99	0.34
CLN3	1.26	0.52

Supplementary figure 4. Assessment of mitochondrial complex IV protein levels and mitochondrial DNA (mtDNA) copy number in CLN3 patient-derived cells. (**A**) Heatmap of proteins belonging to the mitochondrial respiratory chain supercomplex IV in CLN3 patient and control groups at iPSC and astrocyte stages. (**B**) Relative mtDNA copy number was assessed in astrocytes using quantitative PCR (qPCR). Total DNA was extracted, and mtDNA levels were quantified by amplifying the mitochondrial-encoded gene *RNR1,* normalized to the nuclear-encoded reference gene *HBB* (human beta-globin). Data are presented as fold change relative to the nuclear gene for CLN3 patient and control astrocytes. Error bars represent the standard deviation (SD) representing the mean of three technical replicates from two independent biological experiments. Statistical analysis using the student t-test revealed no significant differences between the two groups.

A

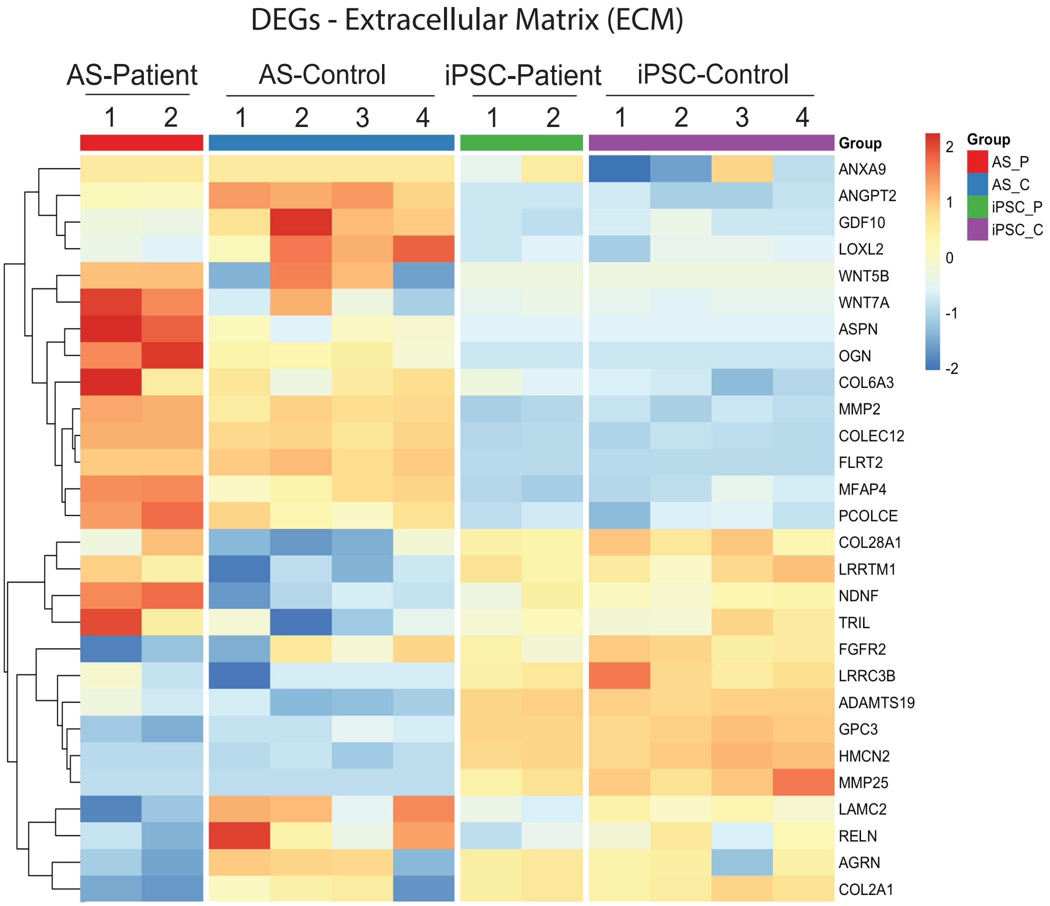

B

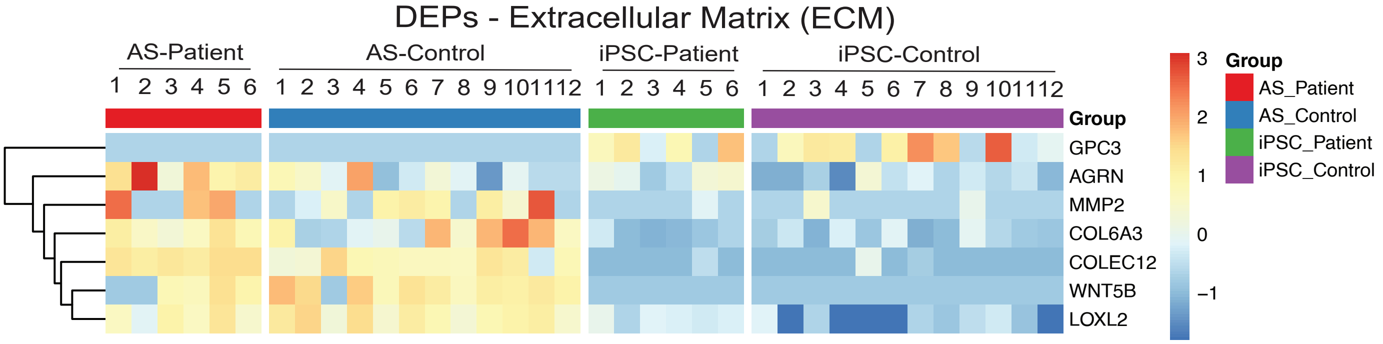

Supplementary figure 5. Heatmap of genes involved in extracellular matrix (ECM) organization in CLN3 patient and control groups at distinct differentiation stages (iPSC and astrocyte). (**A**) mRNA expression levels and (**B**) protein abundance of ECM-associated genes. The number of replicates for each group is indicated, as described in figure 3.

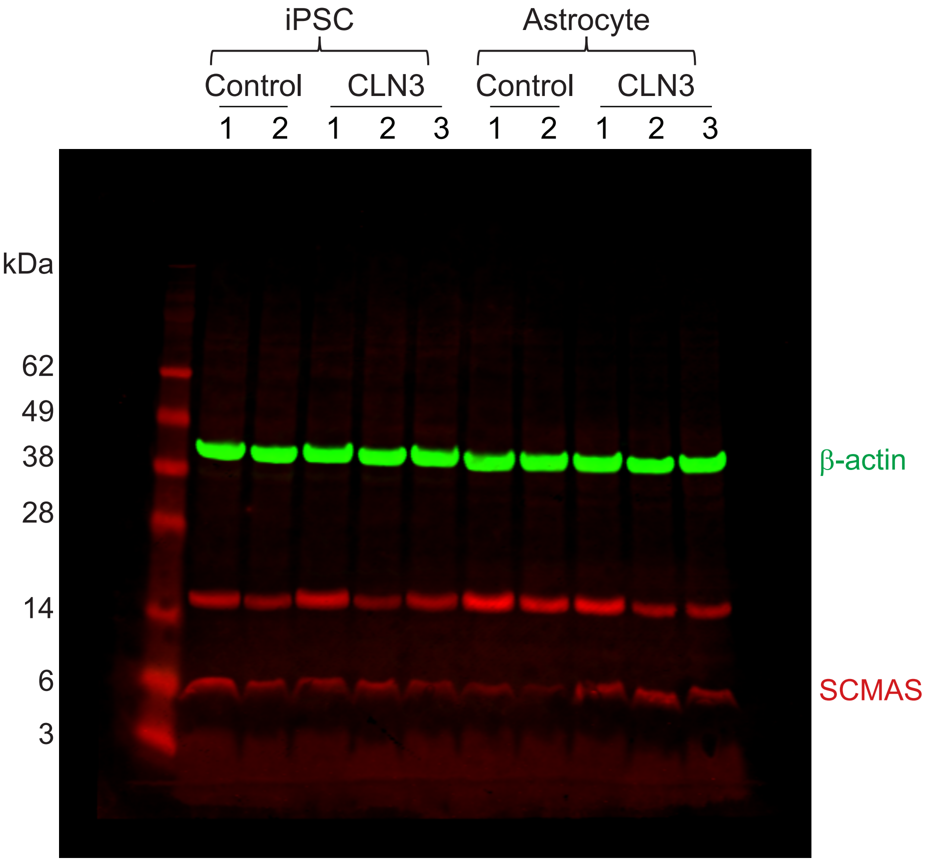

Supplementary figure 6. Uncropped Western blot images shown in figure 3F. Blots were probed for SCMAS (ab181243, abcam) with β-actin (A5441, Sigma) included to confirm equal protein loading across lanes. Molecular weight markers are indicated, and all lanes correspond to patient-derived and control astrocyte samples as described in the main figure. No additional modifications were made to the blot images beyond cropping for presentation in the main figure.
